# p53 induces transcriptional and translational programs to suppress cell proliferation and growth

**DOI:** 10.1186/gb-2013-14-4-r32

**Published:** 2013-04-17

**Authors:** Fabricio Loayza-Puch, Jarno Drost, Koos Rooijers, Rui Lopes, Ran Elkon, Reuven Agami

**Affiliations:** 1 Division of Gene Regulation, The Netherlands Cancer Institute, Plesmanlaan 121, Amsterdam, 1066 CX, The Netherlands

**Keywords:** cell proliferation and growth, mTOR, p53 signaling, ribosome profiling, senescence, translation regulation

## Abstract

**Background:**

Cell growth and proliferation are tightly connected to ensure that appropriately sized daughter cells are generated following mitosis. Energy stress blocks cell growth and proliferation, a critical response for survival under extreme conditions. Excessive oncogenic stress leads to p53 activation and the induction of senescence, an irreversible state of cell-cycle arrest and a critical component in the suppression of tumorigenesis. Nutrient-sensing and mitogenic cues converge on a major signaling node, which regulates the activity of the mTOR kinase. Although transcriptional responses to energy and oncogenic stresses have been examined by many gene-expression experiments, a global exploration of the modulation of mRNA translation in response to these conditions is lacking.

**Results:**

We combine RNA sequencing and ribosomal profiling analyses to systematically delineate modes of transcriptional and translational regulation induced in response to conditions of limited energy, oncogenic stress and cellular transformation. We detect a key role for mTOR and p53 in these distinct physiological states, and provide the first genome-wide demonstration that p53 activation results in mTOR inhibition and a consequent global repression of protein translation. We confirm the role of the direct p53 target genes *Sestrin1 *and *Sestrin2 *in this response, as part of the broad modulation of gene expression induced by p53 activation.

**Conclusions:**

We delineate a bimodal tumor-suppressive regulatory program activated by p53, in which cell-cycle arrest is imposed mainly at the transcriptional level, whereas cell growth inhibition is enforced by global repression of the translation machinery.

## Background

Cell growth (increase in cell mass) and proliferation (increase in cell number) are tightly coupled to ensure that appropriately sized daughter cells are produced after mitosis. In single-cell eukaryotes such as yeast, cell growth and proliferation are mainly regulated by nutrient-sensing pathways. In multicellular organisms, these two processes are also regulated by growth and mitogenic signals, which are integrated with the nutrient-sensing pathways. These nutrient-sensing and mitogenic signals converge on a critical node, which regulates the activity of the highly conserved mTOR kinase [1]. Disregulated cell growth and proliferation are two fundamental aspects of tumorigenesis. It is therefore not surprising that pivotal proto-oncogenes (for example, *RAS, PI3K *and *Akt*) and tumor-suppressor genes (for example, *PTEN, NF1 *and *LKB1*) directly regulate the activity of the mTOR pathway, and that elevated mTOR signaling has been detected in a large proportion of human cancers [2,3]. Consequently, mTOR has emerged as a key target for the treatment of cancer and a number of mTOR inhibitors are being examined by clinical trials [4,5].

A major safeguarding role against cancer development is played by the p53 tumor suppressor [6,7]. Excessive oncogenic signaling ('oncogenic stress') leads to the activation of p53 and to the induction of senescence, an irreversible state of cell-cycle arrest [8,9]. Abrogation of the p53 pathway leads to senescence-bypass and progression to neoplastic transformation [10]. The coupling of cell proliferation and growth signals suggests a role for p53 in controlling cellular growth. However, while the role of p53 in arresting cell proliferation is very well established, its role in arresting cell growth is much less documented. Recent reports described cross-talks between p53 and mTOR pathways [11,12].

Until recently, systems-level analysis of biological processes was mainly limited to the transcriptomic layer. For almost two decades now, gene-expression microarrays have enabled large-scale exploration of transcriptional modulation under various physiological conditions and in response to numerous stresses. By contrast, systematic exploration of the modulation of mRNA translation significantly lagged behind due to the lack of a genomic technique that probes this regulatory layer. Very recently, a deep-sequencing based technique called ribosome profiling, or Ribo-Seq [13,14], was developed. It allows, for the first time, the study - on a truly global scale - of changes in rates of protein translation (Figure S1A in Additional file 1).

In this study we combined RNA-Seq and Ribo-Seq analyses to systematically explore modes of transcriptional and translational control in conditions of limited nutrients (quiescence), oncogenic stress (senescence) and cellular neoplastic transformation. Our results detect major patterns of transcriptional and translational responses induced by these stresses and indicate critical roles for mTOR and p53 in their regulation.

## Results

### Patterns of transcriptional and translational regulation associated with decreased cell growth and proliferation

**Figure 1:**
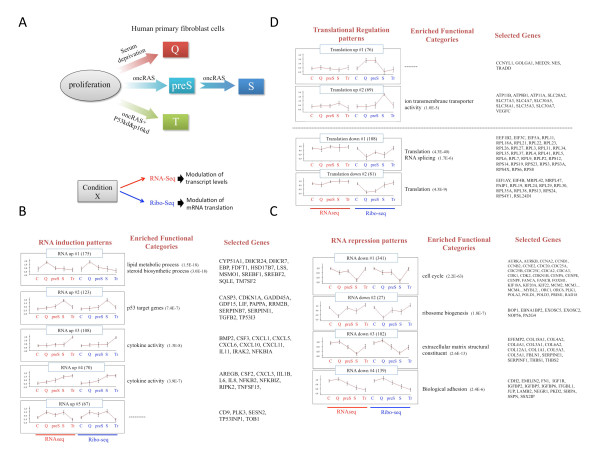
**Wide-scale exploration of patterns of transcriptional and translational regulation in response to energy and oncogenic stresses using RNA-Seq and Ribo-Seq**. **(A) **We profiled gene-expression levels (RNA-Seq) and rates of mRNA translation (Ribo-Seq) in human primary fibroblast BJ cells subjected to serum depletion (energy stress which results in quiescence (Q) or induction of RAS^G12V ^(oncogenic stress which results in senescence (S) in the presence of functional p53, and in neoplastic transformation (T) in the absence of functional p53 and p16^INK4A^). We examined the effect of RAS^G12V ^at two time points, 5 days (pre-senescence; preS) and 14 days (senescence; S). **(B-D) **Transcripts that showed either differential expression or differential translation efficiency (TE) were identified in the combined RNA-Seq and Ribo-Seq dataset and subjected to cluster analysis. Major patterns of RNA induction (B), RNA repression (C) and modulation of TE (D) were detected. Each cluster is represented by the mean pattern of its assigned transcripts (error bars represent ±SD). The first five points (red) represent transcript levels and the subsequent five points (blue) represent mRNA translation levels. Prior to clustering, levels measured for each transcript were standardized to mean = 0 and SD = 1 (so transcripts that are clustered together show similar response pattern in our dataset but might differ in the magnitude of their response). Enriched functional categories (*P*-value calculated using hypergeometric tail) and selected genes are indicated next to the patterns. C, control.

We set out to explore, on genomic and transcriptomic scales, cellular regulation of transcription and translation associated with the modulation of cell growth and proliferation. We therefore applied in parallel RNA-Seq and Ribo-Seq analyses to immortalized human primary BJ fibroblast cells under the following conditions: normal proliferation; quiescence, induced by serum depletion; senescence, induced by activation of the oncogenic *RAS^G12V ^*gene, and examined at early (5 days; herein referred to as pre-senescent state) and late (14 days; fully senescent) time points; and neoplastic transformation, induced by RAS^G12V ^in the background of stable p53 and p16^INK4A ^knockdowns and SV40 small-T expression (Figure 1A). (For details on this transformed cellular system see [10].) Both RNA-Seq and Ribo-Seq measurements showed a high degree of reproducibility (r >0.95) between biological replicates that were measured on the same sequencer run, whereas lower reproducibility was observed between samples measured on different runs. Therefore, each test condition was compared to the control sample (cells grown under normal conditions) of the same batch (Figure S1B,C,D in Additional file 1). In addition, Ribo-Seq reads featured the expected location (markedly depleted from 3′ untranslated regions (UTRs)) and frame distribution (most reads started at frame 0 of the codons) (Table S1 in Additional file 2). The subsequent analyses included 9,686 transcripts covered by at least 40 reads in both the RNA-Seq and Ribo-Seq datasets, in at least one of the examined conditions.

To detect the major patterns of transcriptional and translational regulation in our dataset, we filtered it for transcripts that showed a change in either their expression level or in their translational efficiency (TE) across the examined conditions (Materials and methods; Figure S1E in Additional file 1), and then subjected this set of transcripts to cluster analysis (Figure 1 and Table S2 in Additional file 3). The majority of clusters showed remarkably symmetric responses between the RNA-Seq and Ribo-Seq measurements (Figure 1B,C, annotated red and blue, respectively). The genes assigned to these clusters were regulated at the RNA level (namely, regulated mainly at transcriptional level and, possibly, to some extent also subject to regulation of transcript stability), and they demonstrated the expected mirroring and transmission of transcript level modulation to rates of protein translation. This high correlation between measurements obtained by these very different techniques (RNA-Seq and Ribo-Seq) attests the competence of Ribo-Seq in faithfully recording rates of protein translation.

Analysis of the clusters representing patterns of RNA induction (Figure 1B) showed that transcripts induced specifically in response to nutrient depletion were significantly enriched for genes that function in steroid biosynthesis (Figure 1B, cluster RNA_up#1. The major transcriptional regulators of the genes that function in this metabolic pathway are sterol-regulatory element binding transcription factor 1 and 2 (SREBF1/2) [15]; both were contained in this cluster (transcript levels of SREBF1 and SREBF2 were induced in quiescence by 2.5- and 3.8-fold, respectively), and their induction resulted in a vast up-regulation of enzymes that function along this pathway (Figure S2 in Additional file 1). The induction of the steroid biosynthesis pathway in quiescence is likely aimed at producing endogenous lipids in the absence of their exogenous supply.

Transcripts that were specifically induced in the senescent state were enriched for p53 targets (Figure 1B, cluster RNA_up#2; for example, CDKN1A (p21), GADD45A, TP53I3), demonstrating the strong activation of p53, the key inducer of senescence (for example, p21 was induced by more than 4-fold). Genes related to cytokine activity were over-represented in the gene clusters induced either specifically in the transformed state (Figure 1B, cluster RNA_up#3) or in both the senescent and transformed ones (Figure 1B, cluster RNA_up#4). In our experimental setup, these two states were driven by expressing of RAS^G12V^, which causes cellular 'hyper-function', one manifestation of which is hyper-secretion of inflammatory-related genes [16]. Cluster RNA_up#5 contained genes that were strongly induced both in the quiescent and senescent states (and to a lesser extent in pre-senescence), but were not induced in the transformed one. That is, these genes were induced in the stressed conditions that lead to attenuated proliferation, prominent among them were *Sestrin2 (SESN2*) and *Polo-like kinase 3*.

Four major patterns of RNA repression were detected in our dataset (Figure 1C). The most prominent among them contained more than 340 transcripts that were vigorously repressed in senescence and to a lesser extent in quiescence (Figure 1C, cluster RNA_down#1). This cluster was overwhelmingly enriched for cell-cycle genes, reflecting the block in cell-cycle progression imposed by serum starvation (quiescence) or RAS^G12V ^activation in the presence of functional p53 (senescence). This cluster also reflects how the absence of p53 and p16^INK4A ^completely abrogates the induction of cell-cycle arrest in the face of oncogenic RAS (no repression of cell-cycle genes was observed in the transformed condition). The next cluster contained genes that were repressed in quiescent and to a lesser extent in senescence (Figure 1C, cluster RNA_down#2), and it was significantly enriched for genes that function in ribosome biogenesis, a critical node for regulation of cell growth. Among these genes were *BOP1*, a component of the PeBow complex that is required for pre-ribosome association; *EBNA1BP2*, a nuclear matrix protein that form a dynamic scaffold for ribosome biogenesis in the nucleolus; *NOP56*, which is required for assembly of the 60S ribosomal subunit; and *PA2G4*, which is present in pre-ribosomal ribonucleoprotein complexes and is involved in ribosome assembly and the regulation of intermediate and late steps of rRNA processing. The next clusters contained genes that were repressed in either senescence or the transformed state, and were enriched, respectively, for extracellular matrix and adhesion proteins (Figure 1C, clusters RNA_down#3 and RNA_down#4).

In addition to patterns of transcriptional modulation, the combined RNA-Seq and Ribo-Seq dataset also revealed major patterns of translational modulation (namely, changes in rates of protein translation that do not simply mirror changes occurring at the transcript level) that are associated with the physiological states of quiescence, senescence and transformation (Figure 1D). Two main patterns of induction of TE and two of TE repression were identified. Notably, the clusters of TE repression revealed one of the strongest responses in our dataset: a global repression of the translation of virtually all the ribosomal proteins (of both the large and small ribosome subunits) and of key factors that function in the initiation, elongation and termination steps of protein translation (Figure 1D, clusters Translation_down #1 and #2). This systematic translational repression of ribosomal protein and translation-factor transcripts, which blocks cellular growth, was strongest in quiescence but was also significantly observed in senescence. Importantly, the absence of functional p53 and p16^INK4A ^(the transformed state) did not only abolish the activation of proliferation arrest (Figure 1C, clusters RNA_down#1) but also completely abrogated the activation of the cell-growth arrest program in response to oncogenic stress.

### Two modes of regulation of the translation apparatus

**Figure 2:**
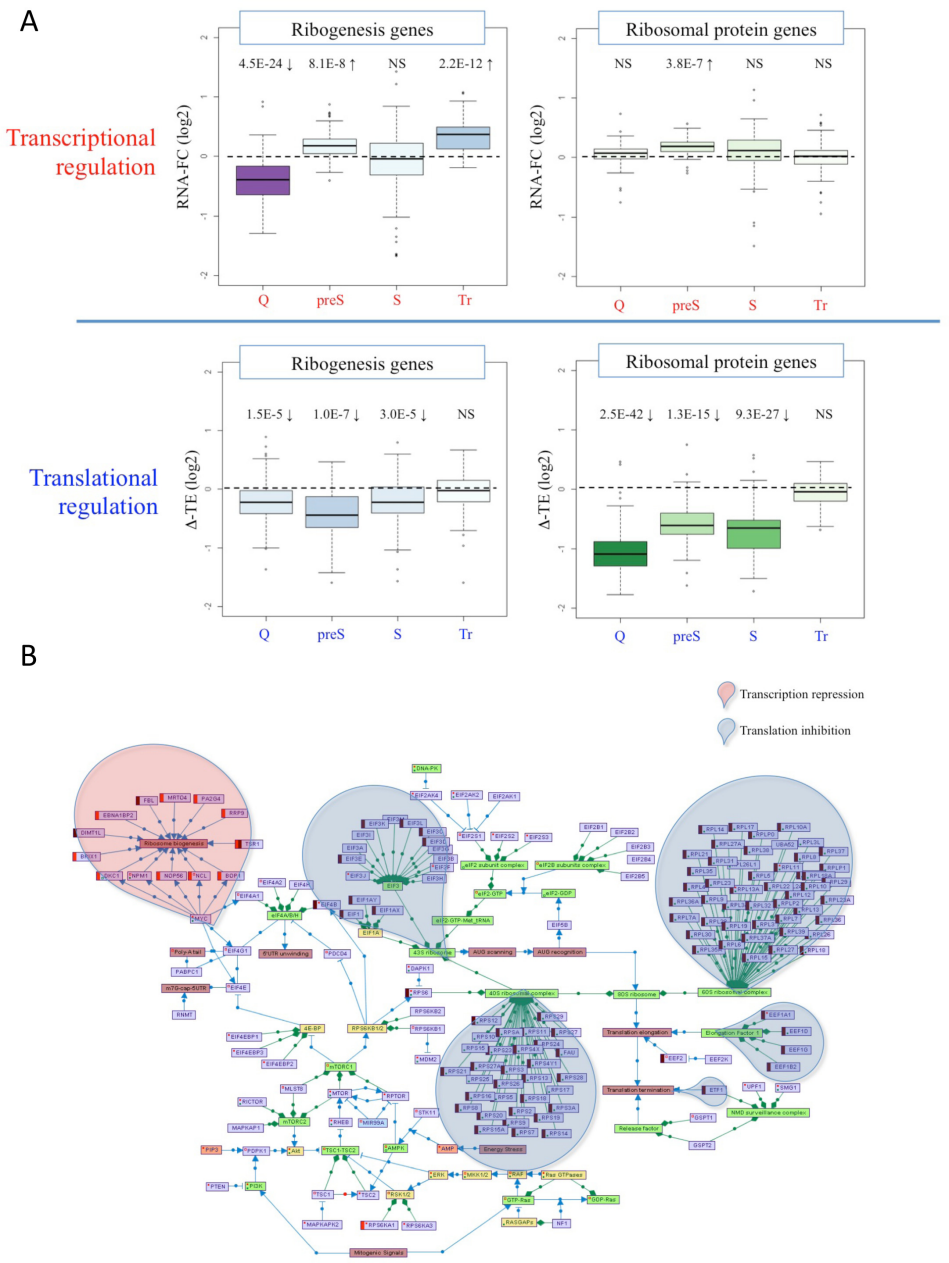
**Two modes of regulation of the translation apparatus**. **(A) **We examined changes in transcript levels (top) and in translation efficiency (TE; bottom), in each condition relative to control proliferating cells, of two functional modules of the translational apparatus: ribogenesis genes (left) and ribosomal protein genes (right). *P*-values were calculated for each condition by comparing changes observed for the test gene-set and for a background set containing all the other transcripts in the dataset; Wilcoxon test. The figure shows that while the ribosomal protein genes were strikingly and exclusively regulated at the layer of mRNA translation, regulation of ribogenesis genes was enforced at the transcriptional level, and to a lesser extent, in some conditions also at the level of translation. **(B) **A comprehensive map of the translational apparatus generated using the SPIKE knowledgebase of signaling pathways. The map contains five types of nodes (violet nodes represent genes/proteins; green, protein complexes; yellow, gene families; pink, biological functions or states (for example, 'translation elongation'); and orange, signaling molecules (for example, AMP)), and two types of edges (blue edges represent regulatory relationship between nodes; arrow for activation and T-shaped edges for inhibition; green edges represent associations between gene families or protein complexes and their members). Genes that were repressed in response to energy stress (quiescence state) in our dataset at the transcriptional or the translational levels are colored, respectively, by orange or brown bars to the left of their nodes. (Red and green dots within nodes indicate that the nodes have additional regulation and association relationships in the in SPIKE database that are not displayed in the map to reduce clutter).

Examination of the major patterns detected in our dataset suggested that, in response to energy stress (serum depletion), the cells activated a double-armed regulatory program to achieve global attenuation of protein synthesis and thereby arrest cell growth. One arm of this program imposed transcriptional repression of genes that function in ribosome biogenesis (for example, rRNA processing and assembly of the ribosome) (Figure 1C, cluster RNA_down#2), while the second arm enforced repression of the translation of the ribosomal proteins themselves and of key translational factors (Figure 1D, clusters Translation_down #1 and #2). To test this hypothesis more systematically, we compared how genes functionally annotated as playing a role in ribosome biogenesis (Gene Ontology (GO) term GO:0042254, 120 genes) and the ribosomal protein genes (99 genes) were regulated in our dataset. This comparison clearly showed a distinct mode of regulation during energy stress: while the ribosomal protein genes were regulated exclusively at the layer of translation (Figure 2A), ribosome-genesis genes were mainly regulated at the transcriptional level.

Next, we used the SPIKE knowledgebase of signaling pathways [17] to build a comprehensive map of the protein-translation apparatus, and used this map to demonstrate the bimodal regulation of the translational machinery in response to energy stress. The two functionally distinct modules of this machinery, comprising the auxiliary ribosome-genesis genes on the one hand and the ribosomal proteins and translation initiation, elongation and termination factors on the other, were clearly regulated at distinct, yet highly coordinated, regulatory layers - the former functional module was mainly regulated at the transcriptional level, whereas the latter was regulated at the mRNA translational level (Figure 2B).

### Translational repression of the translation machinery is a molecular hallmark of mTOR inhibition

Recently, Hsieh *et al*. [18] applied the combined RNA-Seq and Ribo-Seq approach to prostate cancer cells treated with two mTOR inhibitors: rapamycin, which is an allosteric mTOR inhibitor, and PP242, which is a more potent inhibitor that interferes with mTOR's ATP site. Analyzing this dataset, we identified only one major pattern of translation modulation in response to mTOR inhibition. This pattern included more than 150 transcripts whose TE was repressed in response to PP242 and, to a lesser extent, rapamycin (Figure S3A in Additional file 1). This cluster was overwhelmingly enriched for components of the translational apparatus and included virtually all the ribosomal proteins and major translation initiation, elongation and termination factors (Figure S3B in Additional file 1). To statistically examine the effect of mTOR inhibition on the TE of the ribosomal proteins, we compared the change in TE observed for the set of ribosomal protein transcripts to that observed for all the other protein-coding transcripts detected in this dataset. Indeed, ribosomal proteins' TE was strikingly attenuated after treatment with either of the two mTOR inhibitors (Figure S3C in Additional file 1). These results indicate that global translational repression of the cellular translation machinery is a molecular hallmark of mTOR inhibition. This suggests that the comprehensive repression of ribosomal proteins observed in both the quiescent and senescent states (Figure 2A) was mediated through inhibition of the mTOR pathway.

mTOR inhibition in conditions of energy stress is very well established, whereas the inhibition of this pathway in the face of oncogenic stress is much less documented. To gain insights into the mechanism by which the translation of the translational apparatus is regulated, we searched for enriched motifs in the 5′- and 3′-UTR of the transcripts detected in this module. In accordance with previous publications, we found that the 5′-UTRs of these transcripts were significantly enriched for a T/C-rich motif (Figure S3D in Additional file 1), which corresponds to the 5′-terminal oligopyrimidine tract (5′-TOP) element that was previously demonstrated to control the translation of the majority of ribosomal proteins and many key translation factors [19-21].

### p53-mediated attenuation of cell proliferation and growth

**Figure 3:**
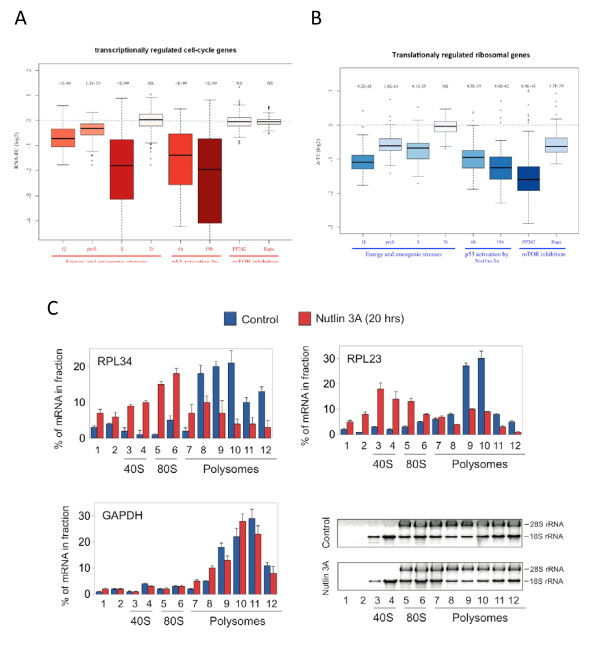
**Bimodal p53-mediated suppression of cell proliferation and growth**. **(A) **Examination of the effect of energy and oncogenic stress, p53 activation by nutlin-3a (6 h and 19 h after treatment) and mTOR inhibition (by PP242 and rapamycin) on the expression level of cell-cycle related transcripts. p53 activation resulted in a very significant attenuation of the expression of cell-cycle genes. *P*-values were calculated for each condition by comparing the distribution of fold-change measured for cell-cycle and all the other genes in the dataset using Wilcoxon test. We included in this analysis a subset of 298 genes selected from the set of GO cell-cycle genes (GO:007049) which showed a variation of at least 1.5-fold in expression level (either induction or repression) across our entire dataset. **(B) **Examination of the effect of energy and oncogenic stress, p53 activation by nutlin-3a and mTOR inhibition on the translation efficiency of the ribosomal protein transcripts. p53 activation resulted in a very significant repression of the translation of ribosomal gene transcripts (*P*-values were calculating as in A). **(C) **Validation of the effect of p53 activation on translation efficiency of ribosomal gene transcripts measured by their ribosome occupancy. MCF-7 cells were treated with nutlin-3a for 20 h and subjected to isolation of polysome-associated mRNAs (see Methods). Polysomal fractionation followed by RT-PCR for two ribosomal genes (and *GAPDH *as a control) were used to quantify mRNA levels in the different polysomal fractions.

While RAS^G12V ^induction in the presence of functional p53 results in senescence, its activation in the background of compromised p53 and p16^INK4A ^leads to the development of neoplastic transformation. As discussed above, our parallel global profiling of transcript and translation levels showed that among the main responses that were imposed by the cells in senescence but not in the transformed state were attenuation of cell-cycle progression (regulated at the layer of transcripts level; Figure 1C, cluster RNA_down#1) and of cell growth (regulated at the layer of mRNA translation; Figure 1D, cluster Translation_down #1). While induction of cell-cycle arrest is one of the most well characterized functions of p53, its role in the regulation of cell growth is less documented. Therefore, we next globally characterized the effect of p53 activation on transcript expression and mRNA translation. We treated immortalized BJ cells with nutlin-3a, an inhibitor of MDM2 and a potent inducer of p53 [22], and applied RNA-Seq and Ribo-Seq to these samples. As expected, nutlin-3a treatment resulted in a very strong induction of a set of p53 target genes (Figure S4 in Additional file 1), and this activation resulted in a sharp down-regulation of the expression of cell-cycle genes (Figure 3A). Most importantly, in addition to modulation of transcript levels, we also revealed that p53 activation resulted in a striking translational repression of the ribosomal proteins and other key translation factors (Figure 3B). We validated this result using standard polysome-fractionation assay followed by RT-PCR of two top regulated ribosomal genes; *RPL34 *and *RPL23*. In contrast to the housekeeping gene *GAPDH*, whose mRNA association with polysomes was not altered following nutlin-3a treatment, both RPL genes showed a clear transcript shift from polysome-associated to ribosome-free fractions (Figure 3C). This result confirms the observed reduced TE of the ribosomal transcripts following p53 activation.

Next, to corroborate our observations and elucidate mechanisms by which p53 affects translation, we examined a second cell line, the MCF-7 breast cancer epithelial cell line that contains wild-type p53. We applied RNA-Seq and Ribo-Seq to examine MCF-7 transcriptional and translational responses to Nulin-3a treatment. As in the case of BJ fibroblasts, p53 activation by nutlin-3a in MCF-7 cells resulted in a transcriptional strong down-regulation of cell-cycle genes and broad translational repression of the ribosomal protein and translation factors (Figure S5 in Additional file 1). Thus, the p53-mediated translational repression of the ribosomal proteins and translation factors seems a broad phenomenon.

**Figure 4:**
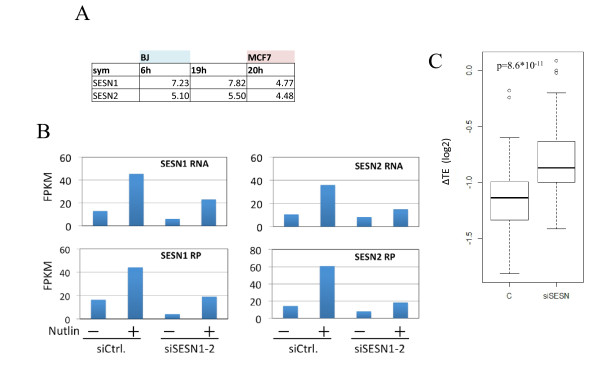
**Knocking-down the Sestrin genes significantly compromises the translational repression of the translation machinery upon p53 activation**. **(A) **A table showing fold induction of *Sestrin1 *and *Sestrin2 (SESN1 *and *SESN2*, respectively) in nutlin-3a treated cells for the indicated times compared to control untreated cells. **(B) **MCF-7 cells were transfected with si-control and si-*SESN1 *and si-*SESN2 *siRNA oligos, treated with nutlin-3a for 20 h, and subjected to RNA-Seq and Ribo-Seq analyses. The effect of siRNAs on the RNA level and ribosome occupancy of *SESN1 *and *SESN2 *is shown. **(C) **Comparison between changes in translation efficiency measured for genes encoding the translational machinery (ribosomal proteins and translation initiation/elongation factors) in response to nutlin-3a treatment, in cells knocked-down for *Sestrin1 *and *Sestrin2 *(right) or treated with control siRNAs (left). *P*-values calculated using Wilcoxon test. FPKM, fragments per kilobase of mRNA per million reads; si, small interfering.

We subsequently sought mechanisms by which p53 exerts its translational-repressive effect. It was previously reported that p53 controls mTOR function through direct activation of *SESN1 *and *SESN2 *[11]. To examine the role of Sestrin-1 and -2 in mediating the translational repression of the translation machinery upon p53 activation, we carried out an RNA-Seq and Ribo-Seq analysis of nutlin-3a-treated and control MCF-7 cells in which both *SESN1 *and *SESN2 *were knocked-down. RNA-Seq and the Ribo-Seq measurements confirmed efficient knockdown of both Sestrin genes (Figure 4B). In line with our expectations, knocking-down the Sestrin genes significantly compromised the p53-induced translational repression of the genes encoding the translation machinery (Figure 4C). Thus, our results pinpoint the Sestrin genes as essential mediators of the p53-mediated global repression of translation, and position mTOR activity in between active p53 and its global effect on the translational machinery.

**Figure 5:**
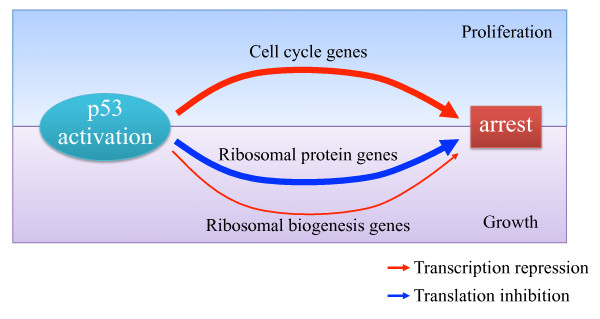
**Schematic model for the bimodal regulatory program activated by p53 to suppress cell growth and proliferation**.

Altogether, our results demonstrate that activation of p53 leads to the simultaneous induction of two tumor-suppressive programs: blocking cell proliferation and arresting cell growth (Figure 5). While the first arm of this bimodal response was strongly detected by the many gene-expression microarray studies that examined p53 responses, the second component was completely overlooked by those studies as it is largely imposed at the layer of translational regulation.

## Discussion

We explored on a genomic- and transcriptomic scale modulation of mRNA levels and their translation rates in physiological conditions of energy deprivation, oncogenic stress and neoplastic transformation. Two major responses that were activated in response to energy and oncogenic stresses but not in the transformed state were the suppression of cell-cycle genes and the inhibition of translational machinery genes. The former represents attenuation of cell proliferation and the latter attenuation of cell growth. Interestingly, while cell-cycle regulation was observed solely at the transcript level, a two-armed program was induced to attenuate protein translation and thereby suppress cell growth. The ribosomal proteins and key translational factors were repressed exclusively at the level of mRNA translation, while the auxiliary genes encoding for proteins that function in rRNA processing and ribosome assembly were mainly down-regulated at the level of transcript expression (Figure 2). In agreement with our observation, a recent study demonstrated a link between mTOR signaling and the transcriptional regulation of ribosome biogenesis genes [23].

Inhibition of the translational machinery is a critical response in the face of stress because protein biosynthesis is the most energy-demanding process in the cell. mTOR is a master regulator of protein synthesis [1], and its inhibition results in global translational repression of the translational machinery (Figure S3 in Additional file 1; [18]). The 5′-UTRs of the translationally repressed transcripts were significantly enriched for the 5′-TOP motif that was demonstrated to control their TE. The mechanisms by which the translation of 5′-TOP transcripts is regulated have remained elusive for a long time and are still under intensive investigation. Recently, Damgaard *et al*. [24] reported that the TIA-1 and TIAR RNA-binding proteins are assembled on the 5′-end of 5′-TOP transcripts in response to serum starvation and that this association, which was dependent on inactivation of the mTOR pathway, blocks the translation of the target transcripts at the initiation step. Thoreen *et al*. [25], however, did not find evidence for the involvement of TIA-1 or TIAR in the regulation of 5′-TOP transcripts, and alternatively suggested that the translation of 5′-TOP mRNAs is especially dependent on the interaction between eIF4G1 and eIF4E initiation factors, which is inhibited by the 4E-BP proteins (see map in Figure 2B). The translation of 5′-TOP mRNAs is enhanced by mTOR-mediated phosphorylation of the 4E-BP inhibitory proteins; in conditions of stress, when mTOR pathway activity is low, 4E-BP proteins bind eIF4E and interfere with its interaction with eIF4G1, thereby selectively attenuating the TE of 5′-TOP transcripts.

Excessive oncogenic signaling activates p53 and induces senescence. Activation of cell-cycle arrest is one of the best characterized tumor-suppressive functions of p53. The observation that both cell-cycle genes and translational machinery transcripts were strongly repressed in senescence (at the transcriptional and translational layers, respectively), but not in the transformed state in which p53 is knocked-down, suggested that p53 activation also strongly inhibits cell growth. We tested this hypothesis by examining the transcriptional and translational responses induced by p53 activation following nutlin-3a treatment. In line with our expectation, p53 activation resulted in a striking translational repression of the translational machinery (Figure 3B). Global translation repression of the translational machinery is a hallmark of mTOR inhibition. This strongly suggests that the repression of the translational machinery upon p53 activation is mediated by inhibition of the mTOR pathway. Supporting this conclusion, we have demonstrated that p53 induction inhibits the phosphorylation of 4E-BP1, a major mTOR target protein. Budanov and Karin [11] reported that two direct targets of p53, Sestrin1 and Sestrin2, mediate p53 inhibition of the mTOR pathway by activating AMP-responsive protein kinase, which is also the main regulator that attenuates mTOR signaling in response to energy stress (see map in Figure 2B). Notably, both Sestrin1 and Sestrin2 were strongly induced in our dataset in response to nutlin-3a treatment, and their inhibition allowed the accumulation of phosphorylated 4E-BP1 in the presence of high p53 levels (Figure 4A). Furthermore, knocking-down the Sestrin genes significantly attenuated the translational repression of the translation machinery in response to p53 activation (Figure 4C). Taken together, our results elucidate, for the first time on a global scale, the extensive impact that p53 activation has on the translation machinery, and demonstrate the role of Sestrin1 and 2 in inhibiting mTOR activity upon p53 activation.

Senescence is usually described as a barrier to tumor development. Recently, Blagosklonny and his colleagues reported that p53 activation paradoxically repressed senescence and converted it into quiescence [26]. A series of follow-up studies demonstrated that the choice between p53-induced senescence and quiescence is determined by the activity of the mTOR pathway, where low mTOR activity results in quiescence and higher activity in senescence [27-31]. Accordingly, Blagosklonny recently sharpened the characterization of the senescent phenotype as a state in which contradicting excessive growth stimulatory and cell-cycle arrest signals coexist in the cell. It is the cell-cycle arrest signals induced by p53 that pose the barrier to tumorigenesis, and not the senescent state *per se *[32]. Our results support this model, and delineate the bimodal regulatory program induced by p53 to enforce concomitant block of both cell proliferation and growth as two coordinated responses that suppress neoplastic transformation.

Our understanding of control mechanisms that translationally co-regulate target mRNAs is scanty and very limited compared to our knowledge on cis-regulatory promoter elements that dictate transcriptional co-regulation of their target genes. The 5′-TOP motif provides one glaring example of a translational co-regulation mechanism. The advent of the Ribo-Seq technique holds great promise for systematic discovery of many more such mechanisms in the coming years, similar to the major advance in the discovery of promoter regulatory elements that followed the maturation of expression arrays more than a decade ago.

## Conclusions

We delineated a bimodal tumor-suppressive regulatory program activated by p53, in which cell-cycle arrest is imposed mainly at the transcriptional level, whereas cell growth inhibition is enforced by global repression of the translation machinery.

## Materials and methods

### Cell culture

Immortalized human BJ primary fibroblast cells (by human telomerase reverse transcriptase expression) were cultured in Dulbecco's modified Eagle's medium supplemented with 10% heat-inactivated fetal calf serum in 5% CO_2 _at 37°C. Retroviruses were made by transient transfection of Ecopack 2 cells (Clontech Laboratories, Inc., Palo Alto, CA, USA) using calcium-phosphate precipitation and harvesting 40 and 64 h later. BJ cells were selected with the proper selection medium 48 h after transduction for at least a week. To obtain pre-senescent and senescent datasets, BJ cells expressing human telomerase reverse transcriptase and tamoxifen-inducible RAS^G12V ^were cultured in the presence of 10^-7 ^M 4-OHT-tamoxifen for 5 and 14 days, respectively. For the transformed dataset, BJ cells expressing human telomerase reverse transcriptase, p16^INK4A^-Knock-Down (KD) p53-KD and SV40 small-T were retrovirally transduced with pBabe-puro-RAS^G12V^. For p53 activation, cells were treated with nutlin-3a (8 mM, Cayman Chemicals, Ann Arbor, MI, USA) for 6 and 19 h. MCF-7 cells were cultured in Dulbecco's modified Eagle's medium supplemented with 10% fetal calf serum. ON-TARGET plus smartPOOL small interfering RNAs (siRNAs) against *SESN1 *and *SESN2 *were purchased from Dharmacon (Lafayette, CO, USA). MCF-7 cells were transfected using Dharmafect 1 reagent (Dharmacon) following the manufacturer's instructions. For inhibition of mTOR, MCF-7 cells were treated with 250 nM of Torin 1 (Tocris Bioscience, Bristol, UK) for 2 h.

### Constructs

pRetrosuper (pRS) was described in [33]. pBabe-puro-RasV12, pBabe-puro-RasV12ERTAM, pMSCV-GFP-st, pBabe-H2B-GFP, pRS-p53 and pRS-p16 were described in [10,34].

### Ribosome profiling (Ribo-Seq)

Cells were treated with cycloheximide (100 μg/ml) for 8 to 10 minutes, washed with ice-cold phosphate-buffered saline (cycloheximide, 100 μg/ml), pelleted, and lysed in buffer A (20 mM Tris-HCl, pH 7.8, 100 mM KCl, 10 mM MgCl2, 1% Triton X-100, 2 mM DTT, 100 μg/ml cycloheximide, 1X complete protease inhibitor). Lysates were centrifuged at 5,000 rpm and the supernatant was treated with 2 U/μl of RNase I (Invitrogen, Grand Island, NY, USA) for 40 min at room temperature. Lysates were fractionated on a linear sucrose gradient (7% to 47%) using the SW-41Ti rotor at 36,000 rpm for 2 h. Fractions enriched in monosomes were pooled and treated with proteinase K (Roche, Mannheim, Germany) in a 1% SDS solution. Released RNA fragments were purified using Trizol reagent and precipitated in the presence of glycogen. For libraries preparation, RNA was gel-purified on a denaturing 10% polyacrylamide urea (7 M) gel. A section corresponding to 30 to 33 nucleotides, the region where most of the ribosome-protected fragments are comprised, was excised, eluted and ethanol precipitated. The resulting fragments were 3′-dephosphorylated using T4 polynucleotide kinase (New England Biolabs Inc. Beverly, MA, USA) for 6 h at 37°C in 2-(N-morpholino)ethanesulfonic acid (MES) buffer (100 mM MES-NaOH, pH 5.5, 10 mM MgCl2, 10 mM β-mercaptoethanol, 300 mM NaCl). 3′ adaptor was added with T4 RNA ligase 1 (New England Biolabs Inc. Beverly, MA, USA) for 2.5 h at 37°C. Ligation products were 5′-phosphorylated with T4 polynucleotide kinase for 30 min at 37°C. 5′ adaptor was added with T4 RNA ligase 1 for 18 h at 22°C.

### Analysis of RNA-Seq and Ribo-Seq datasets

All samples were sequenced using Illumina's HiSeq-2000 platform (Ilumina Inc, San Diago, CA, USA), with read length of 50 nucleotides (see samples definition in Table S1 in Additional file 2; all raw sequence data are deposited at GEO; accession number [GSE:45833]). Sequenced reads were aligned to a reference set of human curated protein-coding transcripts (plus the five human rRNA transcripts) using Bowtie [35]. This reference set was based on Ensembl's gene annotations (release 65). For genes with multiple isoforms, the one with longest coding DNA sequence region and, in case not unique, the one with longest UTR among the ones with the longest coding DNA sequence, was selected to represent the gene. For mapping of RNA-Seq reads, default Bowtie parameters were used with setting -E to 150, which allows up to five mismatches. For Ribo-Seq read mapping, the first 25 nucleotides were used as the 'seed'. Only uniquely mapped reads were used in subsequent analyses. The biological samples that we analyzed together with some global statistics on the alignments are summarized in Table S1 in Additional file 2. As expected, Ribo-Seq reads were markedly depleted from 3′UTRs, and showed characteristic distribution over the transcript reading-frame (Table S1 in Additional file 2). Transcript expression and translation levels were estimated by calculating fragments per kilobase of mRNA per million reads (FPKM) measures per transcript, taking into account either all the reads that map to the transcript (for estimation of expression levels using RNA-Seq data) or only those which map to its coding DNA sequence (for estimation of translation level). FPKM levels below 1.0 were set to 1.0. Both RNA-Seq and Ribo-Seq FPKM measurements were highly reproducible, both showing correlation above 0.95 for biological replicates sequenced on the same sequencer run (Figure S1B in Additional file 1). The correlation between biological replicates processed on different Ribo-Seq runs was lower but still very high (r = 0.82; Figure S1C in Additional file 1). Transcript TE was calculated per condition as the ratio between transcript translation and expression levels (in log2). RNA-Seq and Ribo-Seq data from the study of Hsieh *et al*. [18] that examined responses to mTOR inhibition were downloaded from GEO (accession number [GSE:45833]) and analyzed in the same way.

To detect the major response patterns in our dataset, we first searched for transcripts that showed either differential expression or differential TE in the examined conditions relative to the control proliferating samples. Since we observed a sequencer-run 'batch effect' (Figure S1D in Additional file 1), we compared each test condition to the control sample profiled in the same run (defined in Table S1 in Additional file 2). As variation is larger among lowly expressed transcripts, we set a dynamic cut-off depending on expression level or translation levels (Figure S1E in Additional file 1). A total of approximately 2,800 transcripts passed the cut-off and were subjected to clustering. Clustering and GO enrichment analyses were done using the EXPANDER package [36]. *De novo *motif analysis was done using AMADEUS [37]. All other statistical analyses were done in R.

### Isolation of polysome-associated mRNA

Cells were lysed in buffer A containing 1 U of RnaseOUT (Invitrogen, Grand Island, NY, USA). Lysate was homogenized using a 26 G needle, and the cytosolic extract was obtained by centrifugation at 1,300 g for 10 min. The extract was overlaid on a 7% to 47% linear sucrose gradient and centrifuged in a SW41Ti rotor (Beckman Coulter, California, USA) at 36,000 rpm for 2 h at 4°C. Twelve fractions were collected from the gradients and RNA was isolated from each using Trizol reagent. Reverse transcription was performed using GoScript Reverse Transcription System (Promega, Madison, WI, USA) following the manufacturer's instructions.

### Oligos used

**Library preparation: **3′ adaptor: 5′-AppTCGTATGCCGTCTTCTGCTTG-3′; 5′ adaptor: 5′-GUUCAGAGUUCUACAGUCCGACGAUC-3′.

**RT primer/5′ PCR primer: **5′-CAAGCAGAAGACGGCATA-3′.

**3′ PCR primer: **5′-AATGATACGGCGACCACCGACAGGTTCAGAGTTCTACAGTCCGA-3′.

**Polysome profiling: ***RPS23 *forward: 5'-ACCCTTTTGGAGGTGCTTCT-3'; *RPS23 *reverse: 5'-ATGACCTTTGCGACCAAATC-3'; RPL23 forward: 5'-CTGACAACACAGGAGCCAAA-3'; *RPL23 *reverse: 5'-ACACGCCATCTTTTCTACGG-3'; *RPL34 *forward: 5'-GAGGGGTTCGTGCTGTAAGA-3'; *RPL34 *reverse: 5'-TCTGTGCTTGTGCCTTCAAC-3'.

**qRT-PCR ***SESN1 *forward: 5′-GAGTCTTCGGATGGGTTGAA-3′; *SESN1 *reverse: 5*′*-TGGTCCCTGTCCTAGTGGTC-3′; *SESN2 *forward: 5′-TGCTGTGCTTTGTGGAAGAC-3′; *SESN2 *reverse: 5′-GCTGCCTGGAACTTCTCATC-3*′*

## Abbreviations

FPKM: fragments per kilobase of mRNA per million reads; GO: Gene Ontology; pRS: pRetrosuper; RNA-Seq: RNA sequencing; Ribo-Seq: Ribosome profiling sequencing; siRNA: small interfering RNA; TE: translational efficiency; UTR: untranslated region; 5′-TOP: 5′-terminal oligopyrimidine tract.

## Authors' contributions

FLP, JD and RL performed the experiments. RE and KR analyzed the data. RA supervised the project. RE, FLP and RA wrote the paper. All authors read and approved the final manuscript.

## Supplementary Material

Additional file 1 **Additional figures and legends to support our data**. Click here for file

Additional file 2 **Table S1: Ribo-Seq reads location and frame distribution**. Click here for file

Additional file 3 **Table S2: Genes whose transcripts showed a change in either their expression level or in their translational efficiency across the examined conditions**. Cluster annotation (as in Figure 1) is detailed. Click here for file
