# Supplementary material for: p53 induces transcriptional and translational programs to suppress cell proliferation and growth
Source: Genome Biol. 2013 Apr 17;14(4):R32. doi: 10.1186/gb-2013-14-4-r32 (PMC4053767; doi:10.1186/gb-2013-14-4-r32)
Supplement: Additional file 1 — Additional figures and legends to support our data. [file gb-2013-14-4-r32-S1.PDF]

A

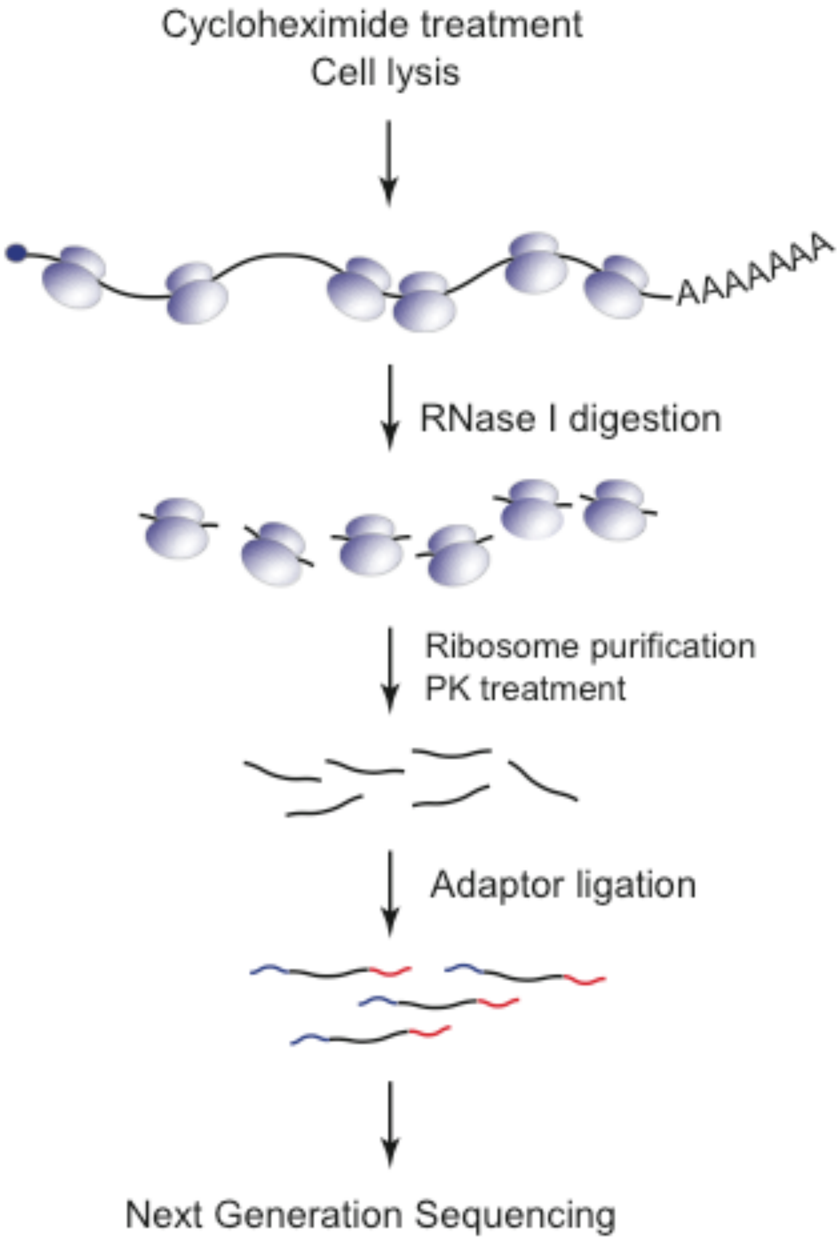
Cycloheximide treatment
Cell lysis
AAAAAAA
RNase I digestion
Ribosome purification
PK treatment
Adaptor ligation
Next Generation Sequencing

Figure S1

B

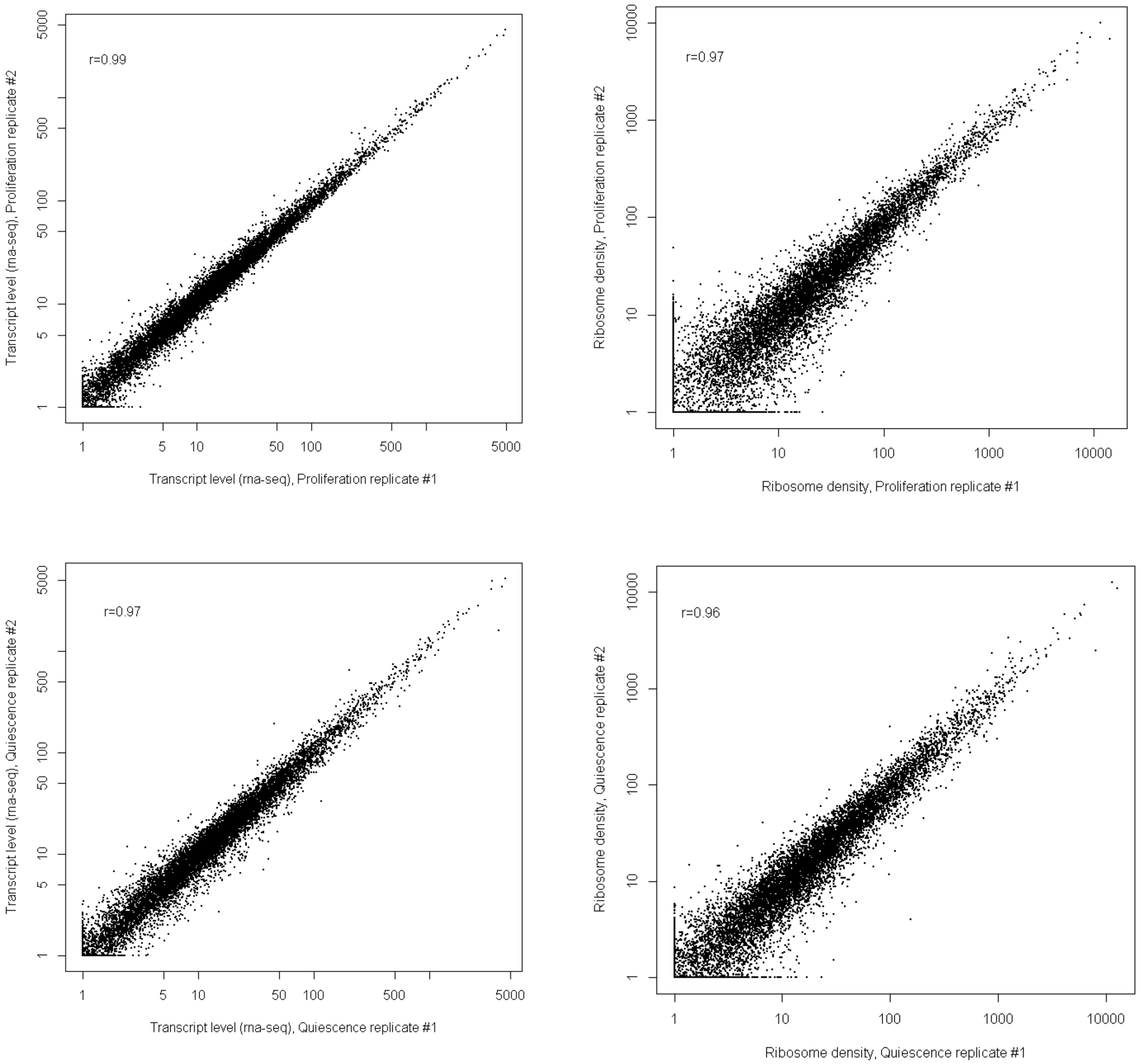
r=0.99
Transcript level (rna-seq), Proliferation replicate #2
Transcript level (rna-seq), Proliferation replicate #1
1
5
10
50
100
500
5000
r=0.97
Ribosome density, Proliferation replicate #2
Ribosome density, Proliferation replicate #1
1
10
100
1000
10000
r=0.97
Transcript level (rna-seq), Quiescence replicate #2
Transcript level (rna-seq), Quiescence replicate #1
r=0.96
Ribosome density, Quiescence replicate #2
Ribosome density, Quiescence replicate #1

Figure S1

C

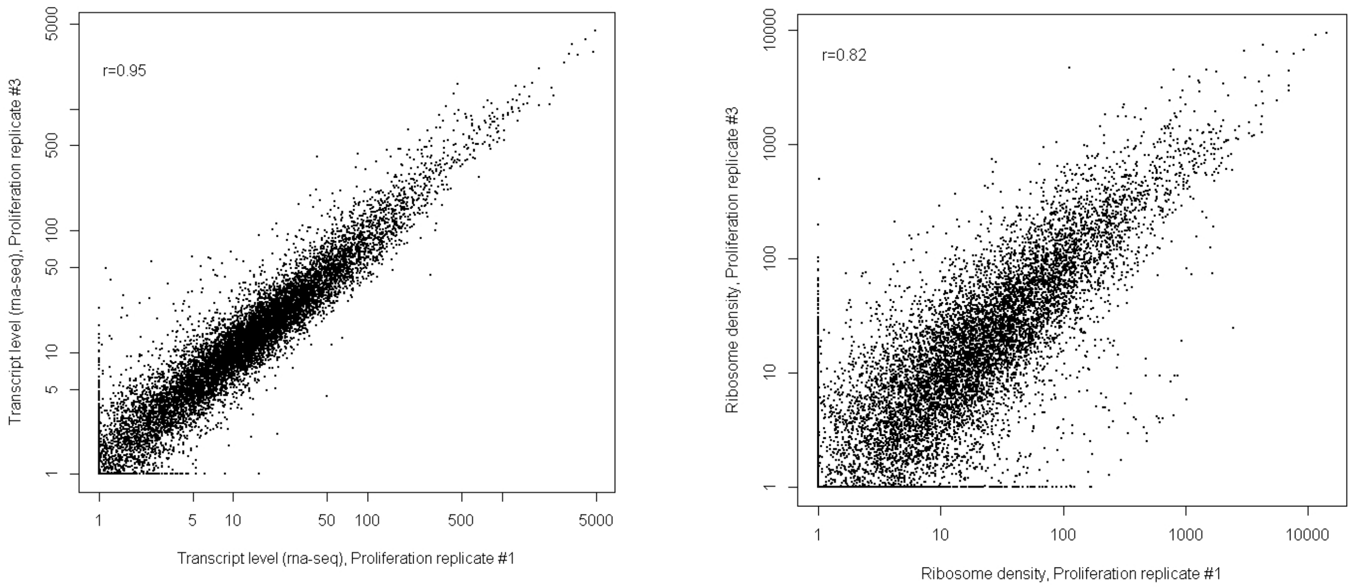
r=0.95
Transcript level (rna-seq), Proliferation replicate #3
Transcript level (rna-seq), Proliferation replicate #1
1
5
10
50
100
500
5000
r=0.82
Ribosome density, Proliferation replicate #3
Ribosome density, Proliferation replicate #1
1
10
100
1000
10000

D

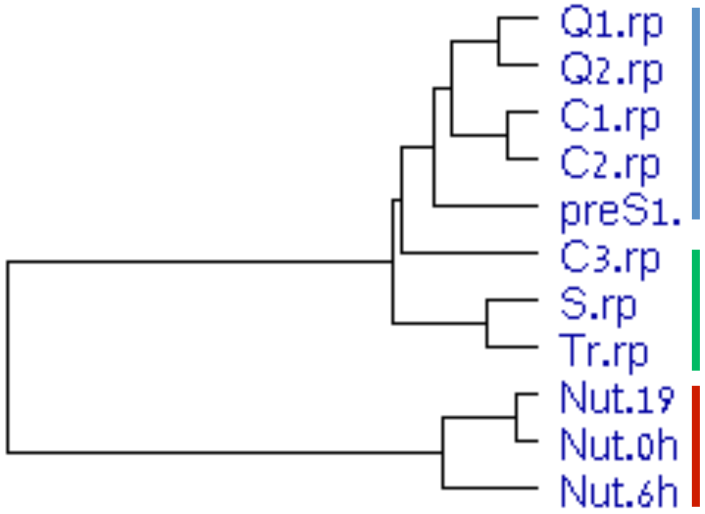
Q1.rp
Q2.rp
C1.rp
C2.rp
preS1.
C3.rp
S.rp
Tr.rp
Nut.19
Nut.0h
Nut.6h

Figure S1

E

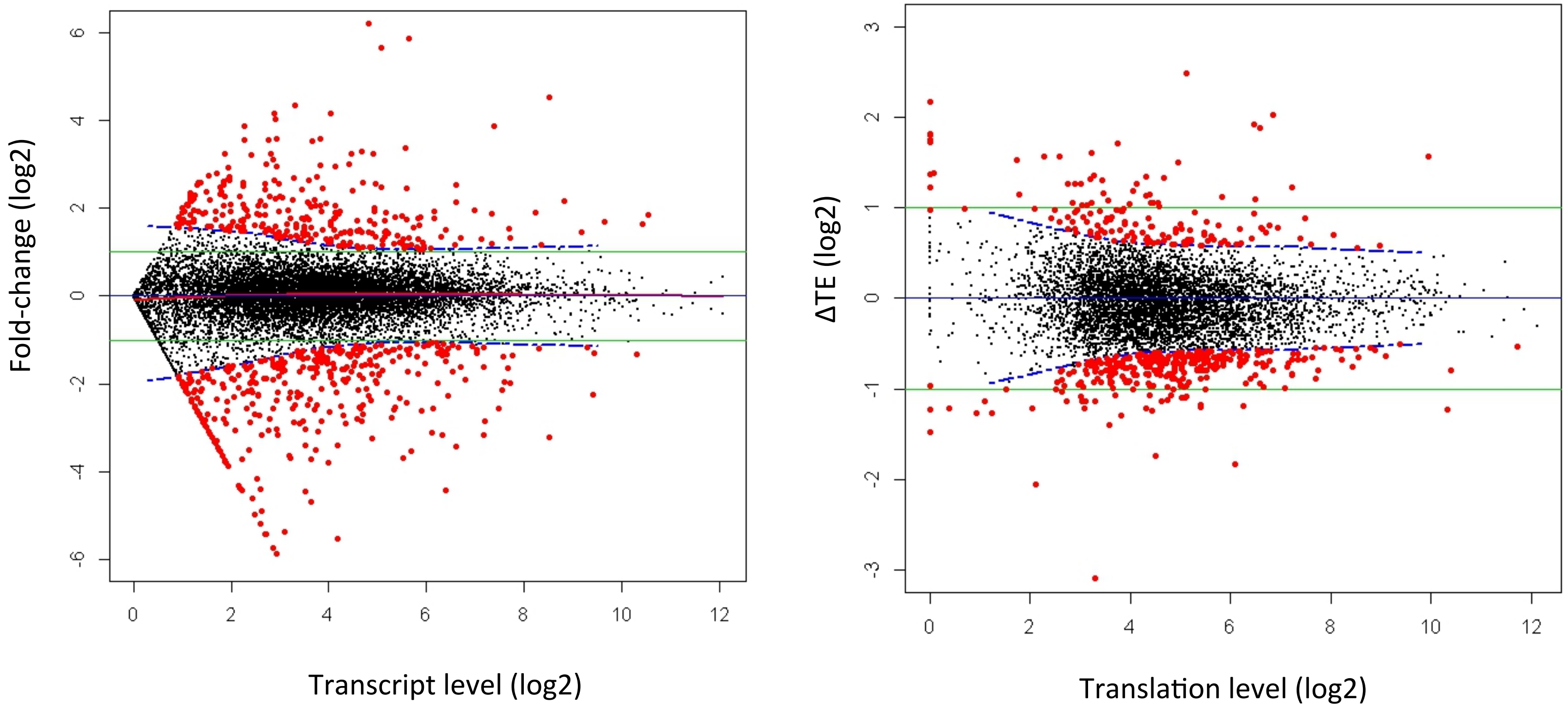
Fold-change (log2)
6
4
2
0
-2
-4
-6
0
2
4
6
8
10
12
Transcript level (log2)
ΔTE (log2)
3
2
1
0
-1
-2
-3
0
2
4
6
8
10
12
Translation level (log2)

Figure S1

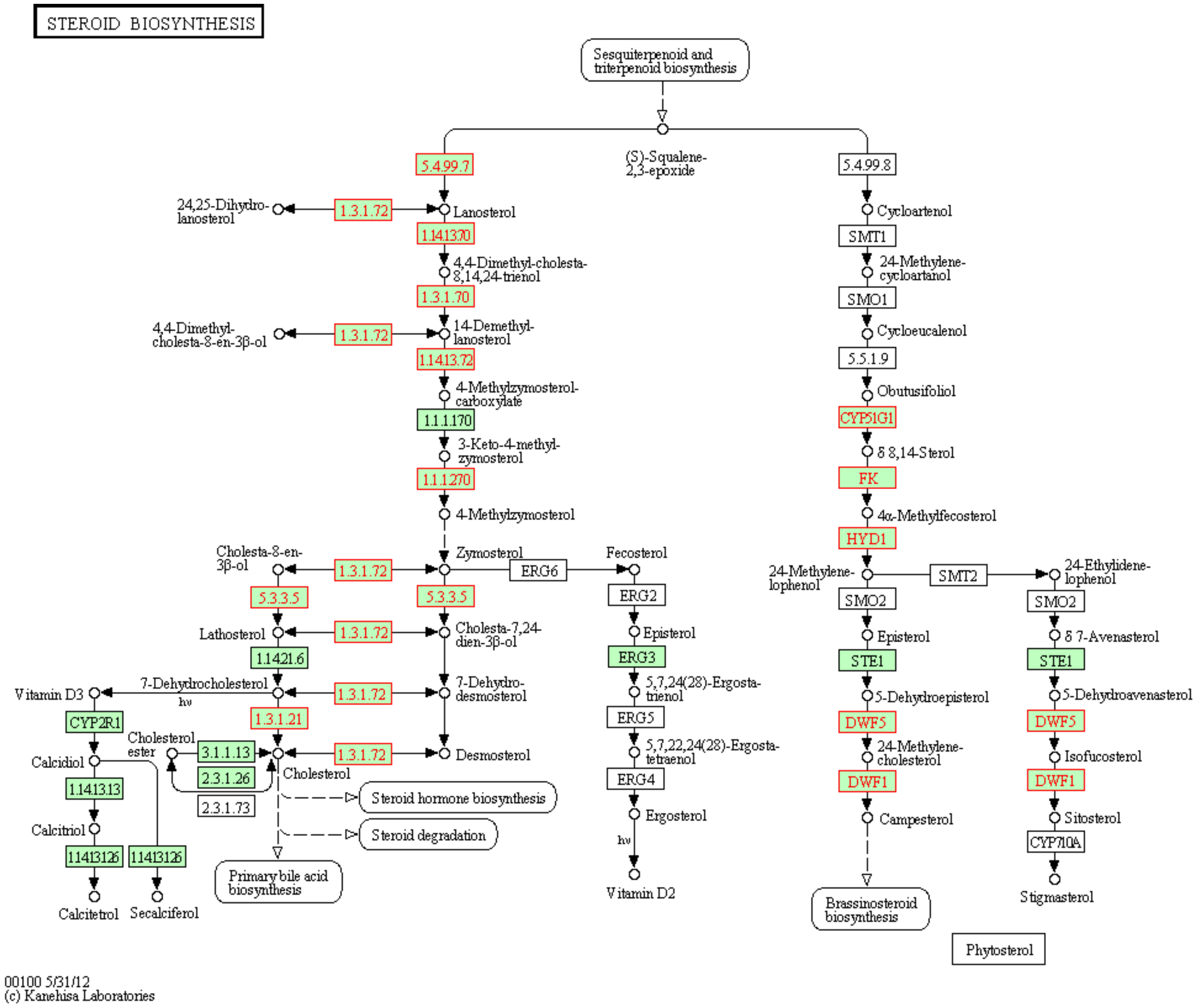
STEROID BIOSYNTHESIS
Sesquiterpenoid and triterpenoid biosynthesis
(S)-Squalene-2,3-epoxide
5.4.99.7
5.4.99.8
24,25-Dihydro-lanosterol
1.3.1.72
Lanosterol
1.14.13.70
4,4-Dimethyl-cholesta-8,14,24-trienol
1.3.1.70
4,4-Dimethyl-cholesta-8-en-3β-ol
1.3.1.72
14-Demethyl-lanosterol
1.14.13.72
4-Methylzymosterol-carboxylate
1.1.1.170
3-Keto-4-methyl-zymosterol
1.1.1.270
4-Methylzymosterol
Cholesta-8-en-3β-ol
1.3.1.72
Zymosterol
ERG6
Fecosterol
5.3.3.5
5.3.3.5
ERG2
Lathosterol
1.3.1.72
Cholesta-7,24-dien-3β-ol
Episterol
1.14.21.6
ERG3
Vitamin D3
7-Dehydrocholesterol
hν
1.3.1.72
7-Dehydro-desmosterol
5,7,24(28)-Ergosta-trienol
CYP2R1
1.3.1.21
ERG5
Cholesterol ester
3.1.1.13
1.3.1.72
Desmosterol
Calcidiol
2.3.1.26
Cholesterol
5,7,22,24(28)-Ergosta-tetraenol
ERG4
1.14.13.13
2.3.1.73
Steroid hormone biosynthesis
Ergosterol
Calcitriol
Steroid degradation
hν
1.14.13.126
1.14.13.126
Primary bile acid biosynthesis
Vitamin D2
Calcitetrol
Secalciferol
Cycloartenol
SMT1
24-Methylene-cycloartanol
SMO1
Cycloeucalenol
5.5.1.9
Obutusifoliol
CYP51G1
δ 8,14-Sterol
FK
4α-Methylfecosterol
HYD1
24-Methylene-lophenol
SMT2
24-Ethylidene-lophenol
SMO2
SMO2
Episterol
δ 7-Avenasterol
STE1
STE1
5-Dehydroepisterol
5-Dehydroavenasterol
DWF5
DWF5
24-Methylene-cholesterol
Isofucosterol
DWF1
DWF1
Campesterol
Sitosterol
CYP710A
Brassinosteroid biosynthesis
Stigmasterol
Phytosterol
00100 5/31/12
(c) Kanehisa Laboratories

Figure S2

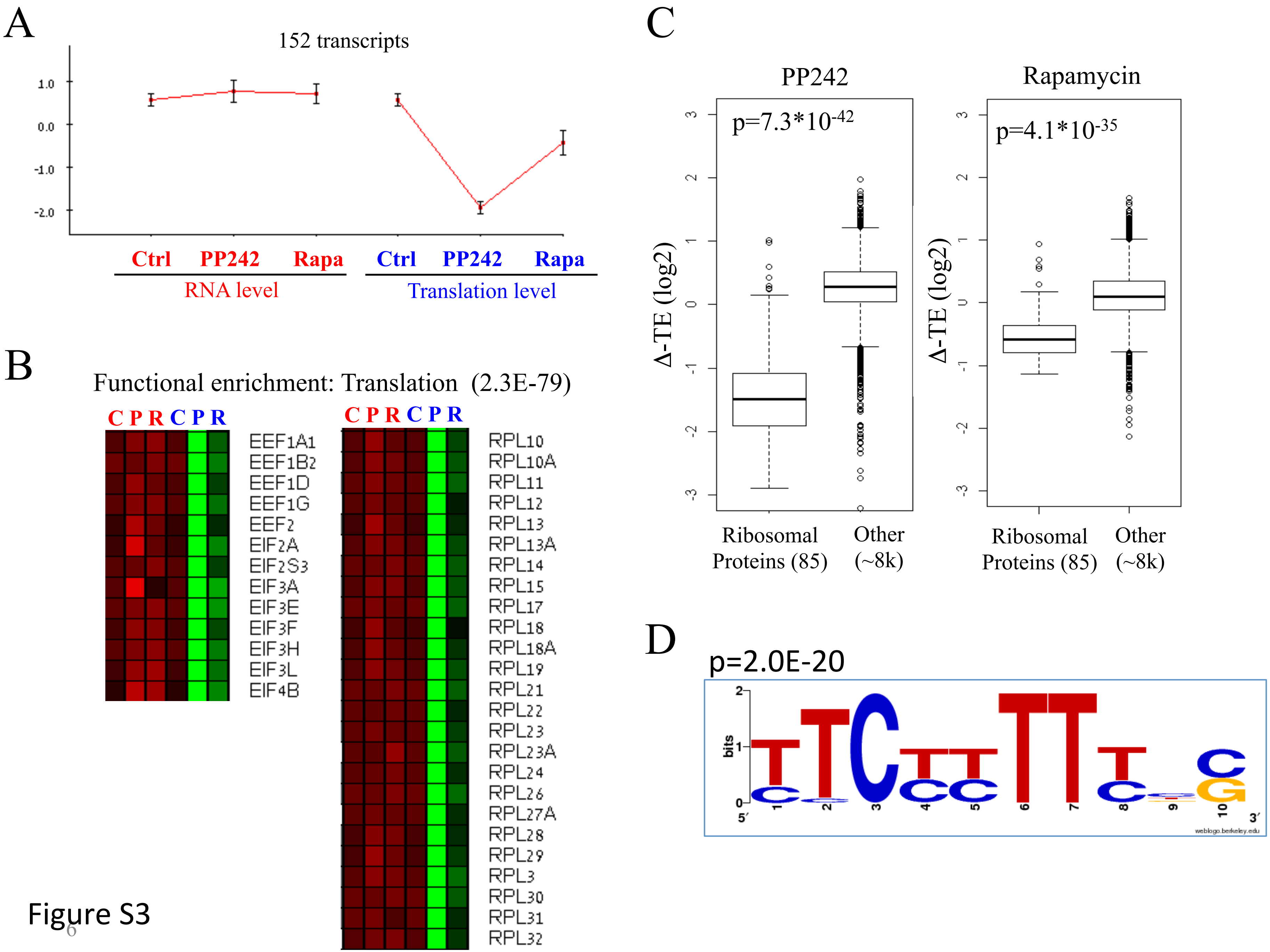

A
152 transcripts
1.0
0.0
-1.0
-2.0
Ctrl PP242 Rapa
RNA level
Ctrl PP242 Rapa
Translation level
B
Functional enrichment: Translation (2.3E-79)
C P R C P R
EEF1A1
EEF1B2
EEF1D
EEF1G
EEF2
EIF2A
EIF2S3
EIF3A
EIF3E
EIF3F
EIF3H
EIF3L
EIF4B
C P R C P R
RPL10
RPL10A
RPL11
RPL12
RPL13
RPL13A
RPL14
RPL15
RPL17
RPL18
RPL18A
RPL19
RPL21
RPL22
RPL23
RPL23A
RPL24
RPL26
RPL27A
RPL28
RPL29
RPL3
RPL30
RPL31
RPL32
C
PP242
p=7.3*10^-42
Rapamycin
p=4.1*10^-35
Δ-TE (log2)
Ribosomal Proteins (85)
Other (~8k)
D
p=2.0E-20
bits
5′
3′
weblogo.berkeley.edu

Figure S3

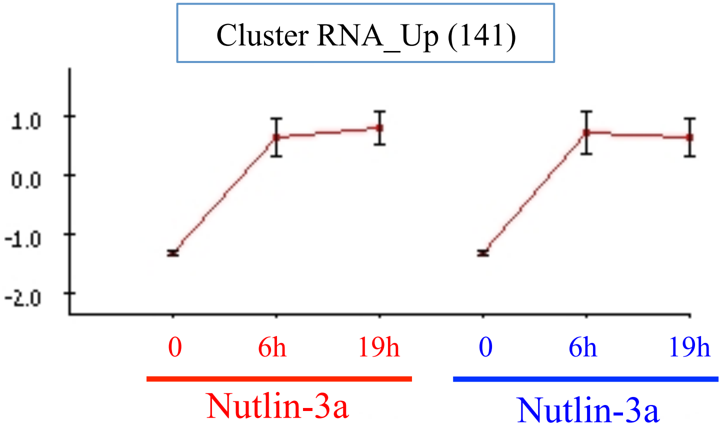

BBC3, BTG2, CCNG1, CDKN1A, DDB2, DRAM1, FBXW7, FUCA1, GDF15, MDM2, NINJ1, PAPPA, PLK3, PPM1D, SERPINE2, SESN1, SESN2, TNFRSF10A, TNFRSF10B, TNFRSF10D, TP53I3, TP53INP1, XPC, ZMAT3, ZNF79

Figure S4

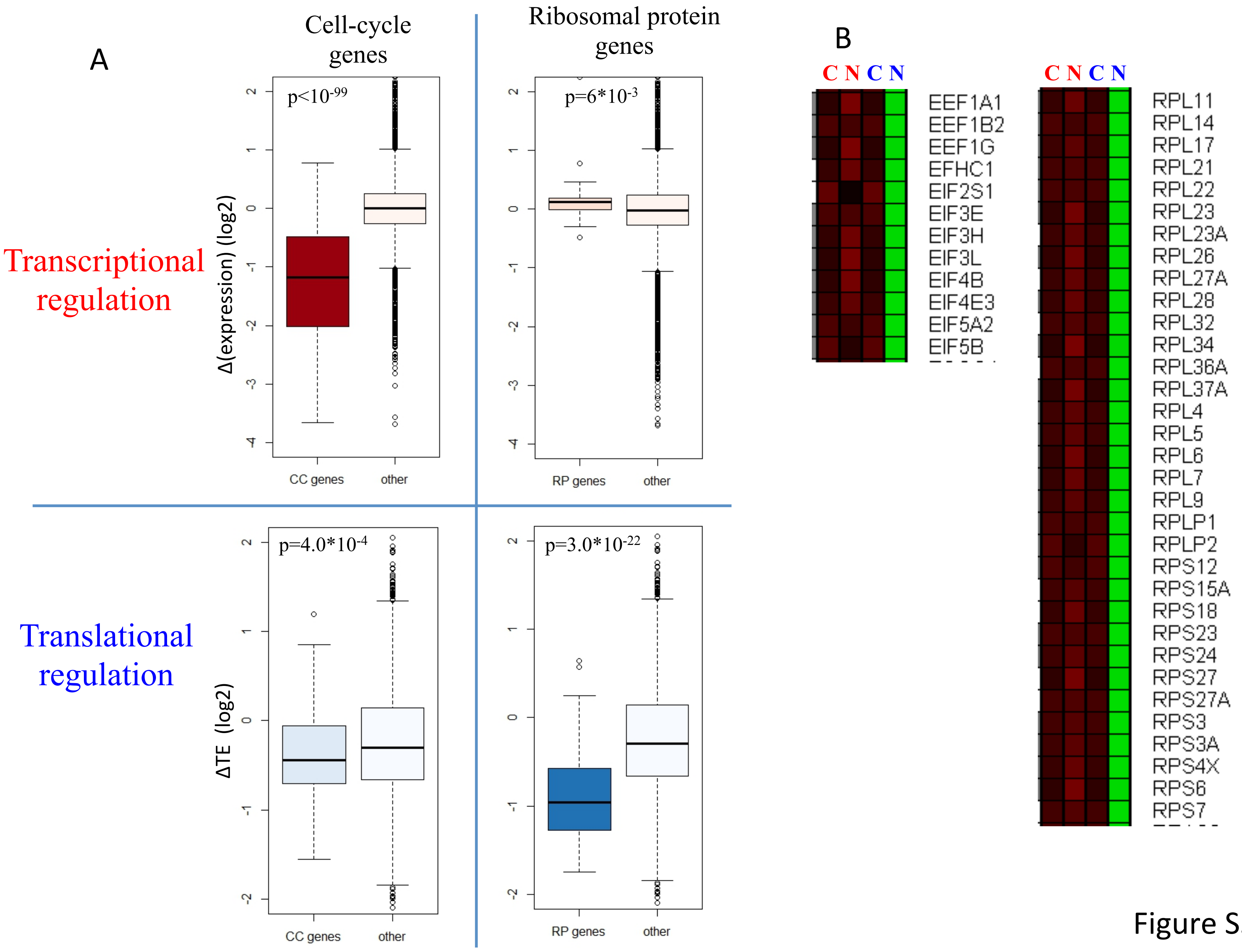
A
Cell-cycle genes
Ribosomal protein genes
Transcriptional regulation
Translational regulation
Δ(expression) (log2)
ΔTE (log2)
p<10-99
p=6*10-3
p=4.0*10-4
p=3.0*10-22
CC genes
other
RP genes
B
C N C N
EEF1A1
EEF1B2
EEF1G
EFHC1
EIF2S1
EIF3E
EIF3H
EIF3L
EIF4B
EIF4E3
EIF5A2
EIF5B
RPL11
RPL14
RPL17
RPL21
RPL22
RPL23
RPL23A
RPL26
RPL27A
RPL28
RPL32
RPL34
RPL36A
RPL37A
RPL4
RPL5
RPL6
RPL7
RPL9
RPLP1
RPLP2
RPS12
RPS15A
RPS18
RPS23
RPS24
RPS27
RPS27A
RPS3
RPS3A
RPS4X
RPS6
RPS7

Figure S5

Additional Figure Legends

Additional Figure 1. Analysis of RNA-Seq and Ribo-Seq data. A. Diagram of ribosome profiling. B. Both transcript expression levels measured by RNA-Seq (left) and transcript translation levels (ribosome density) measured by Ribo-Seq showed very high reproducibility over biological replicates sequenced in the same sequencer run. C. Reproducibility in RNA-Seq and Ribo-Seq measurements was lowered when biological replicates sequenced in different sequencer runs were compared. D. Hierarchical clustering of the Ribo-Seq samples showed a "batch effect" in which samples were mainly separated according to the three processing and sequencing batches in our experiment. Therefore, each test condition was compared to the control sample of the same batch. E. As a filtering step we sought transcripts that showed either differential expression or differential translation-efficiency (TE) across the test samples compared to the control (proliferation under normal conditions) sample. As variability was higher among transcripts with low levels, we calculated a sliding cut-off set to 1.75 local SDs (where SDs were estimated as function of expression (RNA-Seq) or translation (Ribo-Seq) levels). As an example, shown are transcripts that showed differential expression (left) or differential TE (right) in the comparison between the transformed and control samples.

Additional Figure 2. Global induction of the steroid biosynthesis pathway in response to serum depletion. Genes that were specifically induced (at the transcript level) in response to nutrient deprivation were highly enriched (p-value=3.0E-18) for the steroid-biosynthesis pathway. Shown here is the KEGG map for this pathway, having the induced genes colored in red.

Additional Figure 3. mTOR inhibition results in global translational repression of the translation apparatus. A. Combined analysis of RNA-Seq and Ribo-Seq datasets, which profiled responses to two mTOR inhibits, PP242 and rapamycin, detected one cluster of translational modulation. This cluster contained 152 transcripts whose TE was markedly reduced upon mTOR inhibition. Presented is the mean pattern of these transcripts (+/- SD). Prior to clustering, levels measured for each transcript were standardized to mean=0 and SD=1. B. The cluster in A was overwhelmingly enriched for components of the translational apparatus (p-value calculated using hypergeometric tail) and contained virtually all ribosomal-protein genes as well as key initiation/elongation/termination translation factors. C. Comparison between changes in TE measured for ribosomal-protein transcripts and all other transcripts in response to PP242 (left) and rapamycin (right) treatment. (p-values calculated using Wilcoxon test). D. The 5'-UTR sequences of the transcripts repressed by mTOR inhibition were significantly enriched for the T/C-rich 5'-TOP motif.

Additional Figure 4. The cluster of genes that were induced at the transcriptional level by Nutlin-3a treatment (141 genes) was significantly enriched (p-value=2.7E-11) for known direct targets of p53. Shown is the mean pattern (+/- SD) of these genes. Expression and translation levels relative to the control samples were normalized to mean=0 and SD=1 prior to clustering (so the genes clustered together share their response pattern but may differ in their response magnitude). Note the remarkable correlation between transcript and translation levels, reflecting the transmission of the transcriptional activation of these genes to the layer of protein translation. Known p53 targets in this cluster are indicated.

Additional Figure 5. p53 activation in MCF7 cells results too in transcriptional down-regulation of cell-cycle genes and translational repression of ribosomal protein and translation factor genes. A. The change in expression level (top) or translational efficiency (TE; bottom) of the cell-cycle (left) or the ribosomal protein (right) set of genes (same sets as used in Figure 3) was compared with that exhibited by the rest of genes in the MCF7 dataset. (p-values were calculated using Wilcoxon test). B. Heatmap showing the translational factors and ribosomal proteins whose translation was repressed in response to Nutlin-3a treatment of MCF7 cells. (standardized relative levels are shown; C=Control, N=Nutlin-3a treated; The two left samples (red) represent relative expression levels (RNAseq); The two right samples (blue) represent translational rates (RP)).
