# Supplementary material for: p53 induces transcriptional and translational programs to suppress cell proliferation and growth
Source: Genome Biol. 2013 Apr 17;14(4):R32. doi: 10.1186/gb-2013-14-4-r32 (PMC4053767; doi:10.1186/gb-2013-14-4-r32)
Supplement: Additional file 2 — Table S1: Ribo-Seq reads location and frame distribution. [file gb-2013-14-4-r32-S2.PDF]

						Reads mapping to protein-coding transcripts					
	**sample**	**Total Num. of Reads**	**Number of uniquely mapped reads**	**% rRNA**	**Number of non-rRNA reads**	**% 5UTR**	**% CDS**	**% 3UTR**	**% Frame0**	**% Frame 1**	**% Frame 2**
**Ribosome profiling**	C1.rp	147998439	87605975 (59.2%)	90%	8760598	3%	89%	8%	61%	7%	31%
	C2.rp	149411410	103655227 (69.4%)	94%	6219314	4%	84%	12%	60%	6%	34%
	Q1.rp	151488332	113126515 (74.7%)	92%	9050121	3%	87%	10%	73%	6%	21%
	Q2.rp	169525289	103507819 (61.1%)	93%	7245547	4%	83%	13%	65%	14%	21%
	preS.rp	163634462	103483425 (63.2%)	94%	6209006	5%	82%	13%	72%	8%	19%
	C3.rp	161141887	143841524 (89.3%)	92%	11507322	5%	92%	3%	61%	8%	31%
	S.rp	143407219	126190674 (88.0%)	94%	7571440	9%	87%	4%	55%	11%	34%
	Tr.rp	178192873	153110458 (85.9%)	92%	12248837	9%	87%	4%	61%	9%	30%
	C4.rp	166137136	99293757 (59.8%)	88%	11915251	12%	72%	16%	35%	17%	48%
	Nutlin.6h.rp	127701204	88392058 (69.2%)	88%	10607047	13%	79%	9%	37%	16%	47%
	Nutlin.19h.rp	120936988	88229691 (73.0%)	91%	7940672	12%	74%	14%	38%	16%	46%
**RNA-Seq**	C1.rna	26181799	17176883 (65.6%)	3%	16661577	6%	73%	21%	32%	32%	36%
	C2.rna	28243869	18552345 (65.7%)	3%	17995775	6%	72%	22%	32%	32%	36%
	Q1.rna	25495010	16471777 (64.6%)	2%	16142341	6%	71%	23%	32%	32%	36%
	Q2.rna	27075925	17819173 (65.8%)	3%	17284598	6%	72%	22%	32%	32%	36%
	pS1.rna	25117826	17155752 (68.3%)	3%	16641079	6%	72%	22%	32%	32%	36%
	pS2.rna	21619863	14150431 (65.5%)	3%	13725918	6%	72%	22%	32%	32%	36%
	C3.rna (*)	49899303	40892639 (82.0%)	2%	40074786	2%	69%	29%	32%	32%	36%
	S.rna (*)	43395063	34355885 (79.2%)	3%	33325208	2%	68%	30%	32%	32%	36%
	Tr.rna (*)	44581119	35643977 (80.0%)	3%	34574658	3%	68%	29%	32%	32%	36%
	C4.rna	38376326	25777432 (67.2%)	2%	25261883	2%	70%	28%	32%	32%	36%
	Nutlin.6h.rna	43905961	29639414 (67.5%)	2%	29046626	2%	70%	28%	32%	32%	36%
	Nutlin.19h.rna	42308685	28350576 (67.0%)	2%	27783564	2%	70%	28%	32%	32%	36%

- All RNA-Seq samples, except those marked by (*) were paired-end sequenced.
- Samples are colored according to their processing and sequencing runs

Table S1
