# Supplementary material for: p53 induces transcriptional and translational programs to suppress cell proliferation and growth
Source: Genome Biol. 2013 Apr 17;14(4):R32. doi: 10.1186/gb-2013-14-4-r32 (PMC4053767; doi:10.1186/gb-2013-14-4-r32)
Supplement: Additional file 3 — Table S2: Genes whose transcripts showed a change in either their expression level or in their translational efficiency across the examined conditions. Cluster annotation (as in Figure 1) is detailed. [file gb-2013-14-4-r32-S3.PDF]

Table S2

Ensgeneid	Symbol	Cluster
ENSG00000100439	ABHD4	RNA_UP1
ENSG00000131473	ACLY	RNA_UP1
ENSG00000131069	ACSS2	RNA_UP1
ENSG00000117448	AKR1A1	RNA_UP1
ENSG00000136010	ALDH1L2	RNA_UP1
ENSG00000111275	ALDH2	RNA_UP1
ENSG00000109107	ALDOC	RNA_UP1
ENSG00000136859	ANGPTL2	RNA_UP1
ENSG00000154065	ANKRD29	RNA_UP1
ENSG00000196975	ANXA4	RNA_UP1
ENSG00000107282	APBA1	RNA_UP1
ENSG00000189058	APOD	RNA_UP1
ENSG00000196843	ARID5A	RNA_UP1
ENSG00000130707	ASS1	RNA_UP1
ENSG00000054793	ATP9A	RNA_UP1
ENSG00000102409	BEX4	RNA_UP1
ENSG00000134107	BHLHE40	RNA_UP1
ENSG00000104765	BNIP3L	RNA_UP1
ENSG00000122378	C10orf58	RNA_UP1
ENSG00000102760	C13orf15	RNA_UP1
ENSG00000182326	C1S	RNA_UP1
ENSG00000124098	C20orf108	RNA_UP1
ENSG00000164463	C5orf41	RNA_UP1
ENSG00000186577	C6orf1	RNA_UP1
ENSG00000042493	CAPG	RNA_UP1
ENSG00000138764	CCNG2	RNA_UP1
ENSG00000129048	CCRL1	RNA_UP1
ENSG00000156535	CD109	RNA_UP1
ENSG00000182022	CHST15	RNA_UP1
ENSG00000120885	CLU	RNA_UP1
ENSG00000204291	COL15A1	RNA_UP1
ENSG00000158270	COLEC12	RNA_UP1
ENSG00000214575	CPEB1	RNA_UP1
ENSG00000143320	CRABP2	RNA_UP1
ENSG00000165475	CRYL1	RNA_UP1
ENSG00000174080	CTSF	RNA_UP1
ENSG00000178531	CTXN1	RNA_UP1
ENSG00000071967	CYBRD1	RNA_UP1
ENSG00000001630	CYP51A1	RNA_UP1
ENSG00000011465	DCN	RNA_UP1
ENSG00000116133	DHCR24	RNA_UP1
ENSG00000172893	DHCR7	RNA_UP1
ENSG00000147155	EBP	RNA_UP1
ENSG00000170522	ELOVL6	RNA_UP1
ENSG00000138190	EXOC6	RNA_UP1
ENSG00000121769	FABP3	RNA_UP1
ENSG00000134824	FADS2	RNA_UP1
ENSG00000128923	FAM63B	RNA_UP1
ENSG00000111913	FAM65B	RNA_UP1
ENSG00000169710	FASN	RNA_UP1
ENSG00000104870	FCGRT	RNA_UP1
ENSG00000079459	FDFT1	RNA_UP1
ENSG00000160752	FDPS	RNA_UP1
ENSG00000176971	FIBIN	RNA_UP1
ENSG00000176678	FOXL1	RNA_UP1
ENSG00000172020	GAP43	RNA_UP1
ENSG00000131067	GGT7	RNA_UP1
ENSG00000152661	GJA1	RNA_UP1
ENSG00000077585	GPR137B	RNA_UP1
ENSG00000148180	GSN	RNA_UP1
ENSG00000085382	HACE1	RNA_UP1
ENSG00000019991	HGF	RNA_UP1
ENSG00000113161	HMGCR	RNA_UP1
ENSG00000112972	HMGCS1	RNA_UP1
ENSG00000132196	HSD17B7	RNA_UP1
ENSG00000138413	IDH1	RNA_UP1
ENSG00000067064	IDI1	RNA_UP1
ENSG00000185885	IFITM1	RNA_UP1
ENSG00000167779	IGFBP6	RNA_UP1
ENSG00000148798	INA	RNA_UP1
ENSG00000139269	INHBE	RNA_UP1
ENSG00000186480	INSIG1	RNA_UP1
ENSG00000143127	ITGA10	RNA_UP1
ENSG00000130522	JUND	RNA_UP1
ENSG00000084731	KIF3C	RNA_UP1
ENSG00000112769	LAMA4	RNA_UP1
ENSG00000130702	LAMA5	RNA_UP1
ENSG00000213626	LBH	RNA_UP1
ENSG00000130164	LDLR	RNA_UP1
ENSG00000131981	LGALS3	RNA_UP1
ENSG00000138131	LOXL4	RNA_UP1
ENSG00000139679	LPAR6	RNA_UP1
ENSG00000134324	LPIN1	RNA_UP1
ENSG00000177363	LRRN4CL	RNA_UP1
ENSG00000160285	LSS	RNA_UP1
ENSG00000139329	LUM	RNA_UP1
ENSG00000079257	LXN	RNA_UP1
ENSG00000178573	MAF	RNA_UP1
ENSG00000102316	MAGED2	RNA_UP1
ENSG00000166482	MFAP4	RNA_UP1
ENSG00000167578	MIA	RNA_UP1
ENSG00000099953	MMP11	RNA_UP1
ENSG00000198598	MMP17	RNA_UP1
ENSG00000052802	MSMO1	RNA_UP1
ENSG00000038945	MSR1	RNA_UP1
ENSG00000163132	MSX1	RNA_UP1
ENSG00000167508	MVD	RNA_UP1
ENSG00000110921	MVK	RNA_UP1
ENSG00000123933	MXD4	RNA_UP1
ENSG00000119950	MXI1	RNA_UP1
ENSG00000176658	MYO1D	RNA_UP1
ENSG00000104419	NDRG1	RNA_UP1
ENSG00000136999	NOV	RNA_UP1
ENSG00000167799	NUDT8	RNA_UP1
ENSG00000176046	NUPR1	RNA_UP1
ENSG00000182379	NXPH4	RNA_UP1
ENSG00000116774	OLFML3	RNA_UP1
ENSG00000175938	ORAI3	RNA_UP1
ENSG00000135124	P2RX4	RNA_UP1
ENSG00000149090	PAMR1	RNA_UP1
ENSG00000163346	PBXIP1	RNA_UP1
ENSG00000185813	PCYT2	RNA_UP1
ENSG00000150593	PDCD4	RNA_UP1
ENSG00000138735	PDE5A	RNA_UP1
ENSG00000104213	PDGFRL	RNA_UP1
ENSG00000114268	PFKFB4	RNA_UP1
ENSG00000100100	PIK3IP1	RNA_UP1
ENSG00000104368	PLAT	RNA_UP1
ENSG00000187091	PLCD1	RNA_UP1
ENSG00000182836	PLCXD3	RNA_UP1
ENSG00000100344	PNPLA3	RNA_UP1
ENSG00000146278	PNRC1	RNA_UP1
ENSG00000175482	POLD4	RNA_UP1
ENSG00000127948	POR	RNA_UP1
ENSG00000162976	PQLC3	RNA_UP1
ENSG00000126432	PRDX5	RNA_UP1
ENSG00000160013	PTGIR	RNA_UP1
ENSG00000073756	PTGS2	RNA_UP1
ENSG00000163629	PTPN13	RNA_UP1
ENSG00000103485	QPRT	RNA_UP1
ENSG00000072042	RDH11	RNA_UP1
ENSG00000122707	RECK	RNA_UP1
ENSG00000116741	RGS2	RNA_UP1
ENSG00000138835	RGS3	RNA_UP1
ENSG00000099194	SCD	RNA_UP1
ENSG00000112902	SEMA5A	RNA_UP1
ENSG00000149131	SERPING1	RNA_UP1
ENSG00000170624	SGCD	RNA_UP1
ENSG00000198892	SHISA4	RNA_UP1
ENSG00000138771	SHROOM3	RNA_UP1
ENSG00000141469	SLC14A1	RNA_UP1
ENSG00000100075	SLC25A1	RNA_UP1
ENSG00000160326	SLC2A6	RNA_UP1
ENSG00000092068	SLC7A8	RNA_UP1
ENSG00000145335	SNCA	RNA_UP1
ENSG00000124766	SOX4	RNA_UP1
ENSG00000104549	SQLE	RNA_UP1
ENSG00000072310	SREBF1	RNA_UP1
ENSG00000198911	SREBF2	RNA_UP1
ENSG00000164211	STARD4	RNA_UP1
ENSG00000170581	STAT2	RNA_UP1
ENSG00000104946	TBC1D17	RNA_UP1
ENSG00000185339	TCN2	RNA_UP1
ENSG00000178726	THBD	RNA_UP1
ENSG00000149809	TM7SF2	RNA_UP1
ENSG00000170006	TMEM154	RNA_UP1
ENSG00000157111	TMEM171	RNA_UP1
ENSG00000126106	TMEM53	RNA_UP1
ENSG00000125657	TNFSF9	RNA_UP1
ENSG00000109654	TRIM2	RNA_UP1
ENSG00000102804	TSC22D1	RNA_UP1
ENSG00000182704	TSKU	RNA_UP1
ENSG00000184205	TSPYL2	RNA_UP1
ENSG00000111962	UST	RNA_UP1
ENSG00000171724	VAT1L	RNA_UP1
ENSG00000132471	WBP2	RNA_UP1
ENSG00000196998	WDR45	RNA_UP1
ENSG00000070540	WIPI1	RNA_UP1
ENSG00000143816	WNT9A	RNA_UP1
ENSG00000090238	YPEL3	RNA_UP1
ENSG00000119801	YPEL5	RNA_UP1
ENSG00000159714	ZDHHC1	RNA_UP1
ENSG00000162415	ZSWIM5	RNA_UP1
ENSG00000104324		RNA_UP1
ENSG00000188211		RNA_UP1
ENSG00000165029	ABCA1	RNA_UP2
ENSG00000167972	ABCA3	RNA_UP2
ENSG00000162836	ACP6	RNA_UP2
ENSG00000145808	ADAMTS19	RNA_UP2
ENSG00000169252	ADRB2	RNA_UP2
ENSG00000167772	ANGPTL4	RNA_UP2
ENSG00000148677	ANKRD1	RNA_UP2
ENSG00000138772	ANXA3	RNA_UP2
ENSG00000102401	ARMCX3	RNA_UP2
ENSG00000174939	ASPHD1	RNA_UP2
ENSG00000070961	ATP2B1	RNA_UP2
ENSG00000033627	ATP6V0A1	RNA_UP2
ENSG00000185883	ATP6V0C	RNA_UP2
ENSG00000171130	ATP6V0E2	RNA_UP2
ENSG00000133169	BEX1	RNA_UP2
ENSG00000078237	C12orf5	RNA_UP2
ENSG00000168273	C3orf78	RNA_UP2
ENSG00000146409	C6orf192	RNA_UP2
ENSG00000164305	CASP3	RNA_UP2
ENSG00000117335	CD46	RNA_UP2
ENSG00000124762	CDKN1A	RNA_UP2
ENSG00000090661	CERS4	RNA_UP2
ENSG00000250479	CHCHD10	RNA_UP2
ENSG00000166165	CKB	RNA_UP2
ENSG00000137975	CLCA2	RNA_UP2
ENSG00000163347	CLDN1	RNA_UP2
ENSG00000139182	CLSTN3	RNA_UP2
ENSG00000184374	COLEC10	RNA_UP2
ENSG00000101441	CST4	RNA_UP2
ENSG00000135047	CTSL1	RNA_UP2
ENSG00000100612	DHRS7	RNA_UP2
ENSG00000197635	DPP4	RNA_UP2
ENSG00000138166	DUSP5	RNA_UP2
ENSG00000101210	EEF1A2	RNA_UP2
ENSG00000135373	EHF	RNA_UP2
ENSG00000110675	ELMOD1	RNA_UP2
ENSG00000182585	EPGN	RNA_UP2
ENSG00000172031	EPHX4	RNA_UP2
ENSG00000164220	F2RL2	RNA_UP2
ENSG00000145569	FAM105A	RNA_UP2
ENSG00000125848	FLRT3	RNA_UP2
ENSG00000034713	GABARAPL2	RNA_UP2
ENSG00000116717	GADD45A	RNA_UP2
ENSG00000130513	GDF15	RNA_UP2
ENSG00000139278	GLIPR1	RNA_UP2
ENSG00000173221	GLRX	RNA_UP2
ENSG00000196743	GM2A	RNA_UP2
ENSG00000163328	GPR155	RNA_UP2
ENSG00000090339	ICAM1	RNA_UP2
ENSG00000216490	IFI30	RNA_UP2
ENSG00000197081	IGF2R	RNA_UP2
ENSG00000174564	IL20RB	RNA_UP2
ENSG00000008517	IL32	RNA_UP2
ENSG00000259207	ITGB3	RNA_UP2
ENSG00000180332	KCTD4	RNA_UP2
ENSG00000005238	KIAA1539	RNA_UP2
ENSG00000139116	KIF21A	RNA_UP2
ENSG00000203786	KPRP	RNA_UP2
ENSG00000115919	KYNU	RNA_UP2
ENSG00000090530	LEPREL1	RNA_UP2
ENSG00000119862	LGALSL	RNA_UP2
ENSG00000128342	LIF	RNA_UP2
ENSG00000163898	LIPH	RNA_UP2
ENSG00000143353	LYPLAL1	RNA_UP2
ENSG00000143669	LYST	RNA_UP2
ENSG00000140941	MAP1LC3B	RNA_UP2
ENSG00000114738	MAPKAPK3	RNA_UP2
ENSG00000084764	MAPRE3	RNA_UP2
ENSG00000090674	MCOLN1	RNA_UP2
ENSG00000213190	MLLT11	RNA_UP2
ENSG00000196549	MME	RNA_UP2
ENSG00000149968	MMP3	RNA_UP2
ENSG00000165943	MOAP1	RNA_UP2
ENSG00000124532	MRS2	RNA_UP2
ENSG00000172399	MYOZ2	RNA_UP2
ENSG00000049759	NEDD4L	RNA_UP2
ENSG00000204386	NEU1	RNA_UP2
ENSG00000141458	NPC1	RNA_UP2
ENSG00000135318	NT5E	RNA_UP2
ENSG00000173559	OBFC2A	RNA_UP2
ENSG00000119900	OGFRL1	RNA_UP2
ENSG00000123353	ORMDL2	RNA_UP2
ENSG00000081087	OSTM1	RNA_UP2
ENSG00000076641	PAG1	RNA_UP2
ENSG00000157654	PALM2-AKAP2	RNA_UP2
ENSG00000182752	PAPPA	RNA_UP2
ENSG00000184226	PCDH9	RNA_UP2
ENSG00000166908	PIP4K2C	RNA_UP2
ENSG00000123739	PLA2G12A	RNA_UP2
ENSG00000240891	PLCXD2	RNA_UP2
ENSG00000225190	PLEKHM1	RNA_UP2
ENSG00000141682	PMAIP1	RNA_UP2
ENSG00000119938	PPP1R3C	RNA_UP2
ENSG00000143801	PSEN2	RNA_UP2
ENSG00000073008	PVR	RNA_UP2
ENSG00000011478	QPCTL	RNA_UP2
ENSG00000006451	RALA	RNA_UP2
ENSG00000132563	REEP2	RNA_UP2
ENSG00000219200	RNASEK	RNA_UP2
ENSG00000154133	ROBO4	RNA_UP2
ENSG00000048392	RRM2B	RNA_UP2
ENSG00000130066	SAT1	RNA_UP2
ENSG00000073060	SCARB1	RNA_UP2
ENSG00000091490	SEL1L3	RNA_UP2
ENSG00000075223	SEMA3C	RNA_UP2
ENSG00000166396	SERPINB7	RNA_UP2
ENSG00000163536	SERPINI1	RNA_UP2
ENSG00000178537	SLC25A20	RNA_UP2
ENSG00000138821	SLC39A8	RNA_UP2
ENSG00000161011	SQSTM1	RNA_UP2
ENSG00000166900	STX3	RNA_UP2
ENSG00000108639	SYNGR2	RNA_UP2
ENSG00000145022	TCTA	RNA_UP2
ENSG00000092969	TGFB2	RNA_UP2
ENSG00000064115	TM7SF3	RNA_UP2
ENSG00000184988	TMEM106A	RNA_UP2
ENSG00000121895	TMEM156	RNA_UP2
ENSG00000088726	TMEM40	RNA_UP2
ENSG00000173530	TNFRSF10D	RNA_UP2
ENSG00000115129	TP53I3	RNA_UP2
ENSG00000163728	TTC14	RNA_UP2
ENSG00000137267	TUBB2A	RNA_UP2
ENSG00000015532	XYLT2	RNA_UP2
ENSG00000173545	ZNF622	RNA_UP2
ENSG00000197608	ZNF841	RNA_UP2
ENSG00000108798	ABI3	RNA_UP3
ENSG00000154930	ACSS1	RNA_UP3
ENSG00000121281	ADCY7	RNA_UP3
ENSG00000112297	AIM1	RNA_UP3
ENSG00000085662	AKR1B1	RNA_UP3
ENSG00000105290	APLP1	RNA_UP3
ENSG00000148219	ASTN2	RNA_UP3
ENSG00000125845	BMP2	RNA_UP3
ENSG00000167302	C17orf56	RNA_UP3
ENSG00000130775	C1orf38	RNA_UP3
ENSG00000157617	C2CD2	RNA_UP3
ENSG00000182985	CADM1	RNA_UP3
ENSG00000187796	CARD9	RNA_UP3
ENSG00000163814	CDCP1	RNA_UP3
ENSG00000171310	CHST11	RNA_UP3
ENSG00000088882	CPXM1	RNA_UP3
ENSG00000108342	CSF3	RNA_UP3
ENSG00000170373	CST1	RNA_UP3
ENSG00000163739	CXCL1	RNA_UP3
ENSG00000169245	CXCL10	RNA_UP3
ENSG00000169248	CXCL11	RNA_UP3
ENSG00000163735	CXCL5	RNA_UP3
ENSG00000124875	CXCL6	RNA_UP3
ENSG00000161544	CYGB	RNA_UP3
ENSG00000111012	CYP27B1	RNA_UP3
ENSG00000062282	DGAT2	RNA_UP3
ENSG00000140323	DISP2	RNA_UP3
ENSG00000143507	DUSP10	RNA_UP3
ENSG00000142627	EPHA2	RNA_UP3
ENSG00000133216	EPHB2	RNA_UP3
ENSG00000157557	ETS2	RNA_UP3
ENSG00000136379	FAM108C1	RNA_UP3
ENSG00000175182	FAM131A	RNA_UP3
ENSG00000204442	FAM155A	RNA_UP3
ENSG00000042062	FAM65C	RNA_UP3
ENSG00000168672	FAM84B	RNA_UP3
ENSG00000149557	FEZ1	RNA_UP3
ENSG00000179431	FJX1	RNA_UP3
ENSG00000157827	FMNL2	RNA_UP3
ENSG00000172159	FRMD3	RNA_UP3
ENSG00000162654	GBP4	RNA_UP3
ENSG00000107249	GLIS3	RNA_UP3
ENSG00000183840	GPR39	RNA_UP3
ENSG00000189060	H1F0	RNA_UP3
ENSG00000138646	HERC5	RNA_UP3
ENSG00000114315	HES1	RNA_UP3
ENSG00000231389	HLA-DPA1	RNA_UP3
ENSG00000223865	HLA-DPB1	RNA_UP3
ENSG00000149948	HMGA2	RNA_UP3
ENSG00000115756	HPCAL1	RNA_UP3
ENSG00000136720	HS6ST1	RNA_UP3
ENSG00000160223	ICOSLG	RNA_UP3
ENSG00000095752	IL11	RNA_UP3
ENSG00000166578	IQCD	RNA_UP3
ENSG00000134070	IRAK2	RNA_UP3
ENSG00000011201	KAL1	RNA_UP3
ENSG00000162511	LAPTM5	RNA_UP3
ENSG00000136490	LIMD2	RNA_UP3
ENSG00000171517	LPAR3	RNA_UP3
ENSG00000151376	ME3	RNA_UP3
ENSG00000167889	MGAT5B	RNA_UP3
ENSG00000242114	MTFP1	RNA_UP3
ENSG00000182636	NDN	RNA_UP3
ENSG00000165030	NFIL3	RNA_UP3
ENSG00000100906	NFKBIA	RNA_UP3
ENSG00000162711	NLRP3	RNA_UP3
ENSG00000181019	NQO1	RNA_UP3
ENSG00000135114	OASL	RNA_UP3
ENSG00000184588	PDE4B	RNA_UP3
ENSG00000131435	PDLIM4	RNA_UP3
ENSG00000171608	PIK3CD	RNA_UP3
ENSG00000122861	PLAU	RNA_UP3
ENSG00000057657	PRDM1	RNA_UP3
ENSG00000146250	PRSS35	RNA_UP3
ENSG00000117569	PTBP2	RNA_UP3
ENSG00000134247	PTGFRN	RNA_UP3
ENSG00000041353	RAB27B	RNA_UP3
ENSG00000113319	RASGRF2	RNA_UP3
ENSG00000160439	RDH13	RNA_UP3
ENSG00000176641	RNF152	RNA_UP3
ENSG00000147010	SH3KBP1	RNA_UP3
ENSG00000116991	SIPA1L2	RNA_UP3
ENSG00000092096	SLC22A17	RNA_UP3
ENSG00000139508	SLC46A3	RNA_UP3
ENSG00000174640	SLCO2A1	RNA_UP3
ENSG00000101187	SLCO4A1	RNA_UP3
ENSG00000183963	SMTN	RNA_UP3
ENSG00000184557	SOCS3	RNA_UP3
ENSG00000176170	SPHK1	RNA_UP3
ENSG00000166068	SPRED1	RNA_UP3
ENSG00000171621	SPSB1	RNA_UP3
ENSG00000137767	SQRDL	RNA_UP3
ENSG00000152953	STK32B	RNA_UP3
ENSG00000092607	TBX15	RNA_UP3
ENSG00000182134	TDRKH	RNA_UP3
ENSG00000087510	TFAP2C	RNA_UP3
ENSG00000140332	TLE3	RNA_UP3
ENSG00000006118	TMEM132A	RNA_UP3
ENSG00000157600	TMEM164	RNA_UP3
ENSG00000171729	TMEM51	RNA_UP3
ENSG00000146072	TNFRSF21	RNA_UP3
ENSG00000243509	TNFRSF6B	RNA_UP3
ENSG00000124191	TOX2	RNA_UP3
ENSG00000186815	TPCN1	RNA_UP3
ENSG00000104321	TRPA1	RNA_UP3
ENSG00000163874	ZC3H12A	RNA_UP3
ENSG00000171940	ZNF217	RNA_UP3
ENSG00000180884	ZNF792	RNA_UP3
ENSG00000014257	ACPP	RNA_UP4
ENSG00000133805	AMPD3	RNA_UP4
ENSG00000205595	AREGB	RNA_UP4
ENSG00000112276	BVES	RNA_UP4
ENSG00000148426	C10orf47	RNA_UP4
ENSG00000125730	C3	RNA_UP4
ENSG00000196954	CASP4	RNA_UP4
ENSG00000123500	COL10A1	RNA_UP4
ENSG00000164400	CSF2	RNA_UP4
ENSG00000163131	CTSS	RNA_UP4
ENSG00000163734	CXCL3	RNA_UP4
ENSG00000175197	DDIT3	RNA_UP4
ENSG00000145358	DDIT4L	RNA_UP4
ENSG00000128512	DOCK4	RNA_UP4
ENSG00000120875	DUSP4	RNA_UP4
ENSG00000165891	E2F7	RNA_UP4
ENSG00000171617	ENC1	RNA_UP4
ENSG00000115109	EPB41L5	RNA_UP4
ENSG00000124882	EREG	RNA_UP4
ENSG00000178607	ERN1	RNA_UP4
ENSG00000168386	FILIP1L	RNA_UP4
ENSG00000198814	GK	RNA_UP4
ENSG00000165949	IFI27	RNA_UP4
ENSG00000136231	IGF2BP3	RNA_UP4
ENSG00000125538	IL1B	RNA_UP4
ENSG00000136244	IL6	RNA_UP4
ENSG00000169429	IL8	RNA_UP4
ENSG00000164171	ITGA2	RNA_UP4
ENSG00000058085	LAMC2	RNA_UP4
ENSG00000128606	LRRC17	RNA_UP4
ENSG00000065833	ME1	RNA_UP4
ENSG00000166670	MMP10	RNA_UP4
ENSG00000062582	MRPS24	RNA_UP4
ENSG00000119421	NDUFA8	RNA_UP4
ENSG00000067141	NEO1	RNA_UP4
ENSG00000077150	NFKB2	RNA_UP4
ENSG00000144802	NFKBIZ	RNA_UP4
ENSG00000141698	NT5C3L	RNA_UP4
ENSG00000162631	NTNG1	RNA_UP4
ENSG00000164823	OSGIN2	RNA_UP4
ENSG00000145623	OSMR	RNA_UP4
ENSG00000143256	PFDN2	RNA_UP4
ENSG00000087157	PGS1	RNA_UP4
ENSG00000075651	PLD1	RNA_UP4
ENSG00000171680	PLEKHG5	RNA_UP4
ENSG00000128567	PODXL	RNA_UP4
ENSG00000028277	POU2F2	RNA_UP4
ENSG00000108179	PPIF	RNA_UP4
ENSG00000221988	PPT2	RNA_UP4
ENSG00000100994	PYGB	RNA_UP4
ENSG00000154917	RAB6B	RNA_UP4
ENSG00000104312	RIPK2	RNA_UP4
ENSG00000075213	SEMA3A	RNA_UP4
ENSG00000137872	SEMA6D	RNA_UP4
ENSG00000142178	SIK1	RNA_UP4
ENSG00000108932	SLC16A6	RNA_UP4
ENSG00000104635	SLC39A14	RNA_UP4
ENSG00000151012	SLC7A11	RNA_UP4
ENSG00000112096	SOD2	RNA_UP4
ENSG00000115525	ST3GAL5	RNA_UP4
ENSG00000197321	SVIL	RNA_UP4
ENSG00000164362	TERT	RNA_UP4
ENSG00000136891	TEX10	RNA_UP4
ENSG00000249992	TMEM158	RNA_UP4
ENSG00000181634	TNFSF15	RNA_UP4
ENSG00000187688	TRPV2	RNA_UP4
ENSG00000108219	TSPAN14	RNA_UP4
ENSG00000150630	VEGFC	RNA_UP4
ENSG00000121413	ZSCAN18	RNA_UP4
ENSG00000163597		RNA_UP4
ENSG00000123983	ACSL3	RNA_UP5
ENSG00000185567	AHNAK2	RNA_UP5
ENSG00000144746	ARL6IP5	RNA_UP5
ENSG00000126947	ARMCX1	RNA_UP5
ENSG00000027847	B4GALT7	RNA_UP5
ENSG00000164039	BDH2	RNA_UP5
ENSG00000133935	C14orf1	RNA_UP5
ENSG00000198715	C1orf85	RNA_UP5
ENSG00000010278	CD9	RNA_UP5
ENSG00000123989	CHPF	RNA_UP5
ENSG00000147119	CHST7	RNA_UP5
ENSG00000109572	CLCN3	RNA_UP5
ENSG00000110852	CLEC2B	RNA_UP5
ENSG00000161281	COX7A1	RNA_UP5
ENSG00000143387	CTSK	RNA_UP5
ENSG00000159348	CYB5R1	RNA_UP5
ENSG00000128590	DNAJB9	RNA_UP5
ENSG00000256061	DYX1C1-CCPG1	RNA_UP5
ENSG00000170379	FAM115C	RNA_UP5
ENSG00000189320	FAM180A	RNA_UP5
ENSG00000137414	FAM8A1	RNA_UP5

Table S2

ENSG00000179163	FUCA1	RNA_UPS
ENSG00000136542	GALNT5	RNA_UPS
ENSG00000173230	GOLGB1	RNA_UPS
ENSG00000136235	GPNMB	RNA_UPS
ENSG00000152402	GUCY1A2	RNA_UPS
ENSG00000149084	HSD17B12	RNA_UPS
ENSG00000186792	HYAL3	RNA_UPS
ENSG00000125629	INSIG2	RNA_UPS
ENSG00000100196	KDELR3	RNA_UPS
ENSG00000047346	KIAA1370	RNA_UPS
ENSG00000049130	KITLG	RNA_UPS
ENSG00000107798	LIPA	RNA_UPS
ENSG00000111684	LPCAT3	RNA_UPS
ENSG00000154589	LY96	RNA_UPS
ENSG00000109323	MANBA	RNA_UPS
ENSG00000118855	MFSD1	RNA_UPS
ENSG00000204520	MICA	RNA_UPS
ENSG00000147383	NSDHL	RNA_UPS
ENSG00000123240	OPTN	RNA_UPS
ENSG00000130517	PGPEP1	RNA_UPS
ENSG00000173846	PLK3	RNA_UPS
ENSG00000105404	RABAC1	RNA_UPS
ENSG00000042445	RETSAT	RNA_UPS
ENSG00000119729	RHOQ	RNA_UPS
ENSG00000101782	RIOK3	RNA_UPS
ENSG00000109929	SC5DL	RNA_UPS
ENSG00000130766	SESN2	RNA_UPS
ENSG00000168679	SLC16A4	RNA_UPS
ENSG00000119899	SLC17A5	RNA_UPS
ENSG00000110628	SLC22A18	RNA_UPS
ENSG00000166750	SLFN5	RNA_UPS
ENSG00000133138	TBC1D8B	RNA_UPS
ENSG00000166046	TCP11L2	RNA_UPS
ENSG00000135926	TMBIM1	RNA_UPS
ENSG00000181458	TMEM45A	RNA_UPS
ENSG00000141232	TOB1	RNA_UPS
ENSG00000100284	TOM1	RNA_UPS
ENSG00000164938	TP53INP1	RNA_UPS
ENSG00000106537	TSPAN13	RNA_UPS
ENSG00000128311	TST	RNA_UPS
ENSG00000167552	TUBA1A	RNA_UPS
ENSG00000145390	USP53	RNA_UPS
ENSG00000108828	VAT1	RNA_UPS
ENSG00000188706	ZDHHC9	RNA_UPS
ENSG00000108846	ABCC3	RNA_DOWN1
ENSG00000087116	ADAMTS2	RNA_DOWN1
ENSG00000138031	ADCY3	RNA_DOWN1
ENSG00000011426	ANLN	RNA_DOWN1
ENSG00000136938	ANP32B	RNA_DOWN1
ENSG00000143401	ANP32E	RNA_DOWN1
ENSG00000136044	APPL2	RNA_DOWN1
ENSG00000198826	ARHGAP11A	RNA_DOWN1
ENSG00000165801	ARHGEF40	RNA_DOWN1
ENSG00000105011	ASF1B	RNA_DOWN1
ENSG00000066279	ASPM	RNA_DOWN1
ENSG00000156802	ATAD2	RNA_DOWN1
ENSG00000176208	ATAD5	RNA_DOWN1
ENSG00000143153	ATP1B1	RNA_DOWN1
ENSG00000087586	AURKA	RNA_DOWN1
ENSG00000178999	AURKB	RNA_DOWN1
ENSG00000138376	BARD1	RNA_DOWN1
ENSG00000182492	BGN	RNA_DOWN1
ENSG00000089685	BIRC5	RNA_DOWN1
ENSG00000197299	BLM	RNA_DOWN1
ENSG00000136122	BORA	RNA_DOWN1
ENSG00000184992	BRI3BP	RNA_DOWN1
ENSG00000136492	BRIP1	RNA_DOWN1
ENSG00000169679	BUB1	RNA_DOWN1
ENSG00000171792	C12orf32	RNA_DOWN1
ENSG00000185347	C14orf80	RNA_DOWN1
ENSG00000128944	C15orf23	RNA_DOWN1
ENSG00000184220	C3orf26	RNA_DOWN1
ENSG00000205208	C4orf46	RNA_DOWN1
ENSG00000130193	C8orf55	RNA_DOWN1
ENSG00000186193	C9orf140	RNA_DOWN1
ENSG00000153048	CARHSP1	RNA_DOWN1
ENSG00000137812	CASC5	RNA_DOWN1
ENSG00000159231	CBR3	RNA_DOWN1
ENSG00000094916	CBX5	RNA_DOWN1
ENSG00000168502	CCDC165	RNA_DOWN1
ENSG00000040275	CCDC99	RNA_DOWN1
ENSG00000204536	CCHCR1	RNA_DOWN1
ENSG00000145386	CCNA2	RNA_DOWN1
ENSG00000134057	CCNB1	RNA_DOWN1
ENSG00000157456	CCNB2	RNA_DOWN1
ENSG00000175305	CCNE2	RNA_DOWN1
ENSG00000162063	CCNF	RNA_DOWN1
ENSG00000117399	CDC20	RNA_DOWN1
ENSG00000164045	CDC25A	RNA_DOWN1
ENSG00000101224	CDC25B	RNA_DOWN1
ENSG00000158402	CDC25C	RNA_DOWN1
ENSG00000093009	CDC45	RNA_DOWN1
ENSG00000094804	CDC6	RNA_DOWN1
ENSG00000184661	CDCA2	RNA_DOWN1
ENSG00000111665	CDCA3	RNA_DOWN1
ENSG00000170779	CDCA4	RNA_DOWN1
ENSG00000146670	CDCA5	RNA_DOWN1
ENSG00000144354	CDCA7	RNA_DOWN1
ENSG00000164649	CDCA7L	RNA_DOWN1
ENSG00000134690	CDCA8	RNA_DOWN1
ENSG00000170312	CDK1	RNA_DOWN1
ENSG00000123374	CDK2	RNA_DOWN1
ENSG00000111276	CDKN1B	RNA_DOWN1
ENSG00000100526	CDKN3	RNA_DOWN1
ENSG00000167513	CDT1	RNA_DOWN1
ENSG00000115163	CENPA	RNA_DOWN1
ENSG00000138778	CENPE	RNA_DOWN1
ENSG00000117724	CENPF	RNA_DOWN1
ENSG00000153044	CENPH	RNA_DOWN1
ENSG00000123219	CENPK	RNA_DOWN1
ENSG00000100162	CENPM	RNA_DOWN1
ENSG00000166451	CENPN	RNA_DOWN1
ENSG00000138092	CENPO	RNA_DOWN1
ENSG00000031691	CENPQ	RNA_DOWN1
ENSG00000203760	CENPW	RNA_DOWN1
ENSG00000138180	CEP55	RNA_DOWN1
ENSG00000148019	CEP78	RNA_DOWN1
ENSG00000130695	CEP85	RNA_DOWN1
ENSG00000167670	CHAF1A	RNA_DOWN1
ENSG00000159259	CHAF1B	RNA_DOWN1
ENSG00000122966	CIT	RNA_DOWN1
ENSG00000136108	CKAP2	RNA_DOWN1
ENSG00000173207	CKS1B	RNA_DOWN1
ENSG00000123975	CKS2	RNA_DOWN1
ENSG00000166250	CLMP	RNA_DOWN1
ENSG00000128973	CLN6	RNA_DOWN1
ENSG00000092853	CLSPN	RNA_DOWN1
ENSG00000119865	CNRIP1	RNA_DOWN1
ENSG00000103196	CRISPLD2	RNA_DOWN1
ENSG00000137770	CTDSPL2	RNA_DOWN1
ENSG00000107562	CXCL12	RNA_DOWN1
ENSG00000006634	DBF4	RNA_DOWN1
ENSG00000156136	DCK	RNA_DOWN1
ENSG00000013573	DDX11	RNA_DOWN1
ENSG00000124795	DEK	RNA_DOWN1
ENSG00000024526	DEPDC1	RNA_DOWN1
ENSG00000035499	DEPDC1B	RNA_DOWN1
ENSG00000228716	DHFR	RNA_DOWN1
ENSG00000135829	DHX9	RNA_DOWN1
ENSG00000139734	DIAPH3	RNA_DOWN1
ENSG00000126787	DLGAP5	RNA_DOWN1
ENSG00000130816	DNMT1	RNA_DOWN1
ENSG00000113657	DPYSL3	RNA_DOWN1
ENSG00000136982	DSCC1	RNA_DOWN1
ENSG00000143476	DTL	RNA_DOWN1
ENSG00000115380	EFEMP1	RNA_DOWN1
ENSG00000154920	EME1	RNA_DOWN1
ENSG00000213853	EMP2	RNA_DOWN1
ENSG00000186871	ERCC6L	RNA_DOWN1
ENSG00000135476	ESPL1	RNA_DOWN1
ENSG00000174371	EXO1	RNA_DOWN1
ENSG00000123737	EXOSC9	RNA_DOWN1
ENSG00000106462	EZH2	RNA_DOWN1
ENSG00000166801	FAM111A	RNA_DOWN1
ENSG00000189057	FAM111B	RNA_DOWN1
ENSG00000115363	FAM176A	RNA_DOWN1
ENSG00000129195	FAM64A	RNA_DOWN1
ENSG00000101447	FAM83D	RNA_DOWN1
ENSG00000187741	FANCA	RNA_DOWN1
ENSG00000181544	FANCB	RNA_DOWN1
ENSG00000144554	FANCD2	RNA_DOWN1
ENSG00000221829	FANCG	RNA_DOWN1
ENSG00000140525	FANCI	RNA_DOWN1
ENSG00000105202	FBL	RNA_DOWN1
ENSG00000163520	FBLN2	RNA_DOWN1
ENSG00000112029	FBXO5	RNA_DOWN1
ENSG00000168496	FEN1	RNA_DOWN1
ENSG00000096060	FKBP5	RNA_DOWN1
ENSG00000102755	FLT1	RNA_DOWN1
ENSG00000111206	FOXM1	RNA_DOWN1
ENSG00000089280	FUS	RNA_DOWN1
ENSG00000139354	GAS2L3	RNA_DOWN1
ENSG00000117228	GBP1	RNA_DOWN1
ENSG00000092208	GEMIN2	RNA_DOWN1
ENSG00000178295	GEN1	RNA_DOWN1
ENSG00000101003	GINS1	RNA_DOWN1
ENSG00000131153	GINS2	RNA_DOWN1
ENSG00000181938	GINS3	RNA_DOWN1
ENSG00000147536	GINS4	RNA_DOWN1
ENSG00000112312	GMNN	RNA_DOWN1
ENSG00000104518	GSDMD	RNA_DOWN1
ENSG00000177602	GSG2	RNA_DOWN1
ENSG00000075218	GTSE1	RNA_DOWN1
ENSG00000184897	H1FX	RNA_DOWN1
ENSG00000105968	H2AFV	RNA_DOWN1
ENSG00000188486	H2AFX	RNA_DOWN1
ENSG00000092036	HAUS4	RNA_DOWN1
ENSG00000249115	HAUS5	RNA_DOWN1
ENSG00000131351	HAUS8	RNA_DOWN1
ENSG00000149929	HIRIP3	RNA_DOWN1
ENSG00000123485	HJURP	RNA_DOWN1
ENSG00000189403	HMGB1	RNA_DOWN1
ENSG00000164104	HMGB2	RNA_DOWN1
ENSG00000198830	HMGN2	RNA_DOWN1
ENSG00000072571	HMMR	RNA_DOWN1
ENSG00000135486	HNRNPA1	RNA_DOWN1
ENSG00000138668	HNRNPD	RNA_DOWN1
ENSG00000169045	HNRNPH1	RNA_DOWN1
ENSG00000105323	HNRNPUL1	RNA_DOWN1
ENSG00000152795	HNRPDL	RNA_DOWN1
ENSG00000106031	HOXA13	RNA_DOWN1
ENSG00000127483	HP1BP3	RNA_DOWN1
ENSG00000136720	HS6ST1	RNA_DOWN1
ENSG00000178922	HYI	RNA_DOWN1
ENSG00000115738	ID2	RNA_DOWN1
ENSG00000149503	INCENP	RNA_DOWN1
ENSG00000183856	IQGAP3	RNA_DOWN1
ENSG00000142856	ITGB3BP	RNA_DOWN1
ENSG00000197256	KANK2	RNA_DOWN1
ENSG00000178202	KDELC2	RNA_DOWN1
ENSG00000149639	KIAA0889	RNA_DOWN1
ENSG00000103888	KIAA1199	RNA_DOWN1
ENSG00000163507	KIAA1524	RNA_DOWN1
ENSG00000121621	KIF18A	RNA_DOWN1
ENSG00000112984	KIF20A	RNA_DOWN1
ENSG00000079616	KIF22	RNA_DOWN1
ENSG00000137807	KIF23	RNA_DOWN1
ENSG00000142945	KIF2C	RNA_DOWN1
ENSG00000090889	KIF4A	RNA_DOWN1
ENSG00000237649	KIFC1	RNA_DOWN1
ENSG00000149571	KIRREL3	RNA_DOWN1
ENSG00000111057	KRT18	RNA_DOWN1
ENSG00000143815	LBR	RNA_DOWN1
ENSG00000105486	LIG1	RNA_DOWN1
ENSG00000113368	LMNB1	RNA_DOWN1
ENSG00000176619	LMNB2	RNA_DOWN1
ENSG00000164109	MAD2L1	RNA_DOWN1
ENSG00000127241	MASP1	RNA_DOWN1
ENSG00000120539	MASTL	RNA_DOWN1
ENSG00000103495	MAZ	RNA_DOWN1
ENSG00000065328	MCM10	RNA_DOWN1
ENSG00000073111	MCM2	RNA_DOWN1
ENSG00000112118	MCM3	RNA_DOWN1
ENSG00000104738	MCM4	RNA_DOWN1
ENSG00000100297	MCM5	RNA_DOWN1
ENSG00000076003	MCM6	RNA_DOWN1
ENSG00000166508	MCM7	RNA_DOWN1
ENSG00000125885	MCM8	RNA_DOWN1
ENSG00000137337	MDC1	RNA_DOWN1
ENSG00000165304	MELK	RNA_DOWN1
ENSG00000159055	MIS18A	RNA_DOWN1
ENSG00000129534	MIS18BP1	RNA_DOWN1
ENSG00000148773	MKI67	RNA_DOWN1
ENSG00000116062	MSH6	RNA_DOWN1
ENSG00000213347	MXD3	RNA_DOWN1
ENSG00000101825	MXRA5	RNA_DOWN1
ENSG00000101057	MYBL2	RNA_DOWN1
ENSG00000065534	MYLK	RNA_DOWN1
ENSG00000132780	NASP	RNA_DOWN1
ENSG00000010292	NCAPD2	RNA_DOWN1
ENSG00000151503	NCAPD3	RNA_DOWN1
ENSG00000109805	NCAPG	RNA_DOWN1
ENSG00000146918	NCAPG2	RNA_DOWN1
ENSG00000121152	NCAPH	RNA_DOWN1
ENSG00000188566	NDOR1	RNA_DOWN1
ENSG00000109674	NEIL3	RNA_DOWN1
ENSG00000117650	NEK2	RNA_DOWN1
ENSG00000174738	NR1D2	RNA_DOWN1
ENSG00000184162	NR2C2AP	RNA_DOWN1
ENSG00000137404	NRM	RNA_DOWN1
ENSG00000069275	NUCKS1	RNA_DOWN1
ENSG00000143228	NUF2	RNA_DOWN1
ENSG00000137497	NUMA1	RNA_DOWN1
ENSG00000125450	NUP85	RNA_DOWN1
ENSG00000137804	NUSAP1	RNA_DOWN1
ENSG00000104147	OIP5	RNA_DOWN1
ENSG00000085840	ORC1	RNA_DOWN1
ENSG00000091651	ORC6	RNA_DOWN1
ENSG00000165912	PACSIN3	RNA_DOWN1
ENSG00000128050	PAICS	RNA_DOWN1
ENSG00000168078	PBK	RNA_DOWN1
ENSG00000132646	PCNA	RNA_DOWN1
ENSG00000121440	PDZRN3	RNA_DOWN1
ENSG00000178921	PFAS	RNA_DOWN1
ENSG00000067057	PFKP	RNA_DOWN1
ENSG00000077684	PHF17	RNA_DOWN1
ENSG00000119403	PHF19	RNA_DOWN1
ENSG00000127564	PKMYT1	RNA_DOWN1
ENSG00000166851	PLK1	RNA_DOWN1
ENSG00000164087	POC1A	RNA_DOWN1
ENSG00000014138	POLA2	RNA_DOWN1
ENSG00000062822	POLD1	RNA_DOWN1
ENSG00000077514	POLD3	RNA_DOWN1
ENSG00000100479	POLE2	RNA_DOWN1
ENSG00000198901	PRC1	RNA_DOWN1
ENSG00000198056	PRIM1	RNA_DOWN1
ENSG00000154229	PRKCA	RNA_DOWN1
ENSG00000101911	PRPS2	RNA_DOWN1
ENSG00000068489	PRR11	RNA_DOWN1
ENSG00000167157	PRRX2	RNA_DOWN1
ENSG00000164985	PSIP1	RNA_DOWN1
ENSG00000131470	PSMC3IP	RNA_DOWN1
ENSG00000134222	PSRC1	RNA_DOWN1
ENSG00000187514	PTMA	RNA_DOWN1
ENSG00000159335	PTMS	RNA_DOWN1
ENSG00000164611	PTTG1	RNA_DOWN1
ENSG00000161800	RACGAP1	RNA_DOWN1
ENSG00000070950	RAD18	RNA_DOWN1
ENSG00000164754	RAD21	RNA_DOWN1
ENSG00000051180	RAD51	RNA_DOWN1
ENSG00000111247	RAD51AP1	RNA_DOWN1
ENSG00000185379	RAD51D	RNA_DOWN1
ENSG00000197275	RAD54B	RNA_DOWN1
ENSG00000085999	RAD54L	RNA_DOWN1
ENSG00000147274	RBMX	RNA_DOWN1
ENSG00000168476	REEP4	RNA_DOWN1
ENSG00000049541	RFC2	RNA_DOWN1
ENSG00000133119	RFC3	RNA_DOWN1
ENSG00000163918	RFC4	RNA_DOWN1
ENSG00000111445	RFC5	RNA_DOWN1
ENSG00000168411	RFWD3	RNA_DOWN1
ENSG00000159788	RGS12	RNA_DOWN1
ENSG00000143878	RHOB	RNA_DOWN1
ENSG00000175643	RMI2	RNA_DOWN1
ENSG00000104889	RNASEH2A	RNA_DOWN1
ENSG00000167325	RRM1	RNA_DOWN1
ENSG00000171848	RRM2	RNA_DOWN1
ENSG00000141858	SAMD1	RNA_DOWN1
ENSG00000184702	SEPT5	RNA_DOWN1
ENSG00000129810	SGOL1	RNA_DOWN1
ENSG00000130147	SH3BP4	RNA_DOWN1
ENSG00000171241	SHCBP1	RNA_DOWN1
ENSG00000154839	SKA1	RNA_DOWN1
ENSG00000165480	SKA3	RNA_DOWN1
ENSG00000197119	SLC25A29	RNA_DOWN1
ENSG00000125520	SLC2A4RG	RNA_DOWN1
ENSG00000183023	SLC8A1	RNA_DOWN1
ENSG00000072501	SMC1A	RNA_DOWN1
ENSG00000113810	SMC4	RNA_DOWN1
ENSG00000019549	SNAI2	RNA_DOWN1
ENSG00000120896	SORBS3	RNA_DOWN1
ENSG00000076382	SPAG5	RNA_DOWN1
ENSG00000161888	SPC24	RNA_DOWN1
ENSG00000152253	SPC25	RNA_DOWN1
ENSG00000136450	SRSF1	RNA_DOWN1
ENSG00000161547	SRSF2	RNA_DOWN1
ENSG00000112081	SRSF3	RNA_DOWN1
ENSG00000149136	SSRP1	RNA_DOWN1
ENSG00000123473	STIL	RNA_DOWN1
ENSG00000117632	STMN1	RNA_DOWN1
ENSG00000101945	SUV39H1	RNA_DOWN1
ENSG00000078269	SYNJ2	RNA_DOWN1
ENSG00000013810	TACC3	RNA_DOWN1
ENSG00000158710	TAGLN2	RNA_DOWN1
ENSG00000137310	TCF19	RNA_DOWN1
ENSG00000197905	TEAD4	RNA_DOWN1
ENSG00000100726	TELO2	RNA_DOWN1
ENSG00000111602	TIMELESS	RNA_DOWN1
ENSG00000167900	TK1	RNA_DOWN1
ENSG00000166881	TMEM194A	RNA_DOWN1
ENSG00000188807	TMEM201	RNA_DOWN1
ENSG00000120802	TMPO	RNA_DOWN1
ENSG00000132773	TOE1	RNA_DOWN1
ENSG00000160949	TONSL	RNA_DOWN1
ENSG00000163781	TOPBP1	RNA_DOWN1
ENSG00000088325	TPX2	RNA_DOWN1
ENSG00000136527	TRA2B	RNA_DOWN1
ENSG00000183763	TRAIP	RNA_DOWN1
ENSG00000130726	TRIM28	RNA_DOWN1
ENSG00000213186	TRIM59	RNA_DOWN1
ENSG00000071539	TRIP13	RNA_DOWN1
ENSG00000135451	TROAP	RNA_DOWN1
ENSG00000112742	TTK	RNA_DOWN1
ENSG00000176890	TYMS	RNA_DOWN1
ENSG00000175063	UBE2C	RNA_DOWN1
ENSG00000108106	UBE2S	RNA_DOWN1
ENSG00000077152	UBE2T	RNA_DOWN1
ENSG00000012963	UBR7	RNA_DOWN1
ENSG00000034063	UHRF1	RNA_DOWN1
ENSG00000162607	USP1	RNA_DOWN1
ENSG00000119333	WDR34	RNA_DOWN1
ENSG00000160193	WDR4	RNA_DOWN1
ENSG00000075702	WDR62	RNA_DOWN1
ENSG00000092470	WDR76	RNA_DOWN1
ENSG00000166483	WEE1	RNA_DOWN1
ENSG00000109685	WHSC1	RNA_DOWN1
ENSG00000126562	WNK4	RNA_DOWN1
ENSG00000141499	WRAP53	RNA_DOWN1
ENSG00000196584	XRCC2	RNA_DOWN1
ENSG00000126215	XRCC3	RNA_DOWN1
ENSG00000108175	ZMIZ1	RNA_DOWN1
ENSG00000165244	ZNF367	RNA_DOWN1
ENSG00000174442	ZWILCH	RNA_DOWN1
ENSG00000122952	ZWINT	RNA_DOWN1
ENSG00000182319		RNA_DOWN1
ENSG00000167601	AXL	RNA_DOWN2
ENSG00000110330	BIRC2	RNA_DOWN2
ENSG00000170727	BOP1	RNA_DOWN2
ENSG00000112877	CEP72	RNA_DOWN2
ENSG00000143942	CHAC2	RNA_DOWN2
ENSG00000171793	CTPS	RNA_DOWN2
ENSG00000102967	DHODH	RNA_DOWN2
ENSG00000117395	EBNA1BP2	RNA_DOWN2
ENSG00000130713	EXOSC2	RNA_DOWN2
ENSG00000077348	EXOSC5	RNA_DOWN2
ENSG00000172159	FRMD3	RNA_DOWN2
ENSG00000179348	GATA2	RNA_DOWN2
ENSG00000006625	GGCT	RNA_DOWN2
ENSG00000125968	ID1	RNA_DOWN2
ENSG00000117318	ID3	RNA_DOWN2
ENSG00000168685	IL7R	RNA_DOWN2
ENSG00000196497	IPO4	RNA_DOWN2
ENSG00000155090	KLF10	RNA_DOWN2
ENSG00000182481	KPNA2	RNA_DOWN2
ENSG00000160886	LY6K	RNA_DOWN2
ENSG00000101361	NOP56	RNA_DOWN2
ENSG00000170515	PA2G4	RNA_DOWN2
ENSG00000148459	PDSS1	RNA_DOWN2
ENSG00000113356	POLR3G	RNA_DOWN2
ENSG00000176225	RTTN	RNA_DOWN2
ENSG00000075131	TIPIN	RNA_DOWN2
ENSG00000188229	TUBB4B	RNA_DOWN2
ENSG00000106624	AEBP1	RNA_DOWN3
ENSG00000196526	AFAP1	RNA_DOWN3
ENSG00000132746	ALDH3B2	RNA_DOWN3
ENSG00000169083	AR	RNA_DOWN3
ENSG00000134986	C5orf13	RNA_DOWN3
ENSG00000138172	CALHM2	RNA_DOWN3
ENSG00000005059	CCDC109B	RNA_DOWN3
ENSG00000123080	CDKN2C	RNA_DOWN3
ENSG00000181072	CHRM2	RNA_DOWN3
ENSG00000153721	CNKSR3	RNA_DOWN3
ENSG00000111799	COL12A1	RNA_DOWN3
ENSG00000182871	COL18A1	RNA_DOWN3
ENSG00000108821	COL1A1	RNA_DOWN3
ENSG00000168542	COL3A1	RNA_DOWN3
ENSG00000187498	COL4A1	RNA_DOWN3
ENSG00000134871	COL4A2	RNA_DOWN3
ENSG00000130635	COL5A1	RNA_DOWN3
ENSG00000080573	COL5A3	RNA_DOWN3
ENSG00000142173	COL6A2	RNA_DOWN3
ENSG00000163359	COL6A3	RNA_DOWN3
ENSG00000144810	COL8A1	RNA_DOWN3
ENSG00000140848	CPNE2	RNA_DOWN3
ENSG00000109625	CPZ	RNA_DOWN3
ENSG00000184371	CSF1	RNA_DOWN3
ENSG00000175215	CTDSP2	RNA_DOWN3
ENSG00000164932	CTHRC1	RNA_DOWN3
ENSG00000171604	CXXC5	RNA_DOWN3
ENSG00000135929	CYP27A1	RNA_DOWN3
ENSG00000107984	DKK1	RNA_DOWN3
ENSG00000050165	DKK3	RNA_DOWN3
ENSG00000092964	DPYSL2	RNA_DOWN3
ENSG00000172638	EFEMP2	RNA_DOWN3
ENSG00000013016	EHD3	RNA_DOWN3
ENSG00000197594	ENPP1	RNA_DOWN3
ENSG00000136960	ENPP2	RNA_DOWN3
ENSG00000177096	FAM109B	RNA_DOWN3
ENSG00000077942	FBLN1	RNA_DOWN3
ENSG00000151876	FBXO4	RNA_DOWN3
ENSG00000183386	FHL3	RNA_DOWN3
ENSG00000141756	FKBP10	RNA_DOWN3
ENSG00000196924	FLNA	RNA_DOWN3
ENSG00000157240	FZD1	RNA_DOWN3
ENSG00000155760	FZD7	RNA_DOWN3
ENSG00000180447	GAS1	RNA_DOWN3
ENSG00000117226	GBP3	RNA_DOWN3
ENSG00000135821	GLUL	RNA_DOWN3
ENSG00000020181	GPR124	RNA_DOWN3
ENSG00000121957	GPSM2	RNA_DOWN3
ENSG00000138796	HADH	RNA_DOWN3
ENSG00000108840	HDAC5	RNA_DOWN3
ENSG00000094631	HDAC6	RNA_DOWN3
ENSG00000165868	HSPA12A	RNA_DOWN3
ENSG00000126803	HSPA2	RNA_DOWN3
ENSG00000146674	IGFBP3	RNA_DOWN3
ENSG00000140044	JDP2	RNA_DOWN3
ENSG00000107902	LHPP	RNA_DOWN3
ENSG00000115318	LOXL3	RNA_DOWN3
ENSG00000139263	LRIG3	RNA_DOWN3
ENSG00000162591	MEGF6	RNA_DOWN3
ENSG00000011028	MRC2	RNA_DOWN3
ENSG00000008441	NFIX	RNA_DOWN3
ENSG00000166741	NNMT	RNA_DOWN3
ENSG00000175745	NR2F1	RNA_DOWN3
ENSG00000164920	OSR2	RNA_DOWN3
ENSG00000185630	PBX1	RNA_DOWN3
ENSG00000240184	PCDHGC3	RNA_DOWN3
ENSG00000106333	PCOLCE	RNA_DOWN3
ENSG00000113721	PDGFRB	RNA_DOWN3
ENSG00000187800	PEAR1	RNA_DOWN3
ENSG00000092621	PHGDH	RNA_DOWN3
ENSG00000110697	PITPNM1	RNA_DOWN3
ENSG00000184304	PRKD1	RNA_DOWN3
ENSG00000112655	PTK7	RNA_DOWN3
ENSG00000105894	PTN	RNA_DOWN3
ENSG00000168077	SCARA3	RNA_DOWN3
ENSG00000162512	SDC3	RNA_DOWN3
ENSG00000143416	SELENBP1	RNA_DOWN3
ENSG00000135919	SERPINE2	RNA_DOWN3
ENSG00000132386	SERPINF1	RNA_DOWN3
ENSG00000172716	SLFN11	RNA_DOWN3
ENSG00000120833	SOCS2	RNA_DOWN3
ENSG00000159164	SV2A	RNA_DOWN3
ENSG00000089225	TBX5	RNA_DOWN3
ENSG00000196628	TCF4	RNA_DOWN3
ENSG00000111077	TENC1	RNA_DOWN3
ENSG00000105329	TGFB1	RNA_DOWN3
ENSG00000140682	TGFB1I1	RNA_DOWN3
ENSG00000137801	THBS1	RNA_DOWN3

Table S2

ENSG00000186340	THBS2	RNA_DOWN3
ENSG00000162542	TMCO4	RNA_DOWN3
ENSG00000183160	TMEM119	RNA_DOWN3
ENSG00000184584	TMEM173	RNA_DOWN3
ENSG00000253304	TMEM200B	RNA_DOWN3
ENSG00000109084	TMEM97	RNA_DOWN3
ENSG00000082512	TRAF5	RNA_DOWN3
ENSG00000179981	TSHZ1	RNA_DOWN3
ENSG00000068724	TTC7A	RNA_DOWN3
ENSG00000107731	UNC5B	RNA_DOWN3
ENSG00000140836	ZFHX3	RNA_DOWN3
ENSG00000152518	ZFP36L2	RNA_DOWN3
ENSG00000198816	ZNF358	RNA_DOWN3
ENSG00000228594		RNA_DOWN3
ENSG00000107796	ACTA2	RNA_DOWN4
ENSG00000149451	ADAM33	RNA_DOWN4
ENSG00000170214	ADRA1B	RNA_DOWN4
ENSG00000171873	ADRA1D	RNA_DOWN4
ENSG00000162433	AK4	RNA_DOWN4
ENSG00000165092	ALDH1A1	RNA_DOWN4
ENSG00000136010	ALDH1L2	RNA_DOWN4
ENSG00000029534	ANK1	RNA_DOWN4
ENSG00000084234	APLP2	RNA_DOWN4
ENSG00000150347	ARID5B	RNA_DOWN4
ENSG00000145246	ATP10D	RNA_DOWN4
ENSG00000122870	BICC1	RNA_DOWN4
ENSG00000102760	C13orf15	RNA_DOWN4
ENSG00000119280	C1orf198	RNA_DOWN4
ENSG00000182326	C1S	RNA_DOWN4
ENSG00000137463	C4orf49	RNA_DOWN4
ENSG00000181751	C5orf30	RNA_DOWN4
ENSG00000091986	CCDC80	RNA_DOWN4
ENSG00000129048	CCRL1	RNA_DOWN4
ENSG00000156535	CD109	RNA_DOWN4
ENSG00000170558	CDH2	RNA_DOWN4
ENSG00000128656	CHN1	RNA_DOWN4
ENSG00000117519	CNN3	RNA_DOWN4
ENSG00000084636	COL16A1	RNA_DOWN4
ENSG00000164692	COL1A2	RNA_DOWN4
ENSG00000158270	COLEC12	RNA_DOWN4
ENSG00000143320	CRABP2	RNA_DOWN4
ENSG00000109861	CTSC	RNA_DOWN4
ENSG00000071967	CYBRD1	RNA_DOWN4
ENSG00000008256	CYTH3	RNA_DOWN4
ENSG00000011465	DCN	RNA_DOWN4
ENSG00000153904	DDAH1	RNA_DOWN4
ENSG00000134574	DDB2	RNA_DOWN4
ENSG00000162733	DDR2	RNA_DOWN4
ENSG00000104936	DMPK	RNA_DOWN4
ENSG00000115325	DOK1	RNA_DOWN4
ENSG00000142197	DOPEY2	RNA_DOWN4
ENSG00000188641	DPYD	RNA_DOWN4
ENSG00000115468	EFHD1	RNA_DOWN4
ENSG00000120690	ELF1	RNA_DOWN4
ENSG00000132205	EMILIN2	RNA_DOWN4
ENSG00000151491	EPS8	RNA_DOWN4
ENSG00000111913	FAM65B	RNA_DOWN4
ENSG00000078098	FAP	RNA_DOWN4
ENSG00000064763	FAR2	RNA_DOWN4
ENSG00000166147	FBN1	RNA_DOWN4
ENSG00000134285	FKBP11	RNA_DOWN4
ENSG00000079150	FKBP7	RNA_DOWN4
ENSG00000122642	FKBP9	RNA_DOWN4
ENSG00000115414	FN1	RNA_DOWN4
ENSG00000176678	FOXL1	RNA_DOWN4
ENSG00000174804	FZD4	RNA_DOWN4
ENSG00000164574	GALNT10	RNA_DOWN4
ENSG00000136542	GALNT5	RNA_DOWN4
ENSG00000122694	GLIPR2	RNA_DOWN4
ENSG00000155324	GRAMD3	RNA_DOWN4
ENSG00000166923	GREM1	RNA_DOWN4
ENSG00000198189	HSD17B11	RNA_DOWN4
ENSG00000142798	HSPG2	RNA_DOWN4
ENSG00000182054	IDH2	RNA_DOWN4
ENSG00000140443	IGF1R	RNA_DOWN4
ENSG00000115457	IGFBP2	RNA_DOWN4
ENSG00000115461	IGFBP5	RNA_DOWN4
ENSG00000167779	IGFBP6	RNA_DOWN4
ENSG00000109452	INPP4B	RNA_DOWN4
ENSG00000115232	ITGA4	RNA_DOWN4
ENSG00000198542	ITGBL1	RNA_DOWN4
ENSG00000096433	ITPR3	RNA_DOWN4
ENSG00000173801	JUP	RNA_DOWN4
ENSG00000080709	KCNN2	RNA_DOWN4
ENSG00000171345	KRT19	RNA_DOWN4
ENSG00000221852	KRTAP1-5	RNA_DOWN4
ENSG00000196569	LAMA2	RNA_DOWN4
ENSG00000112769	LAMA4	RNA_DOWN4
ENSG00000130702	LAMA5	RNA_DOWN4
ENSG00000172037	LAMB2	RNA_DOWN4
ENSG00000183722	LHFP	RNA_DOWN4
ENSG00000064042	LIMCH1	RNA_DOWN4
ENSG00000071282	LMCD1	RNA_DOWN4
ENSG00000136153	LMO7	RNA_DOWN4
ENSG00000163431	LMOD1	RNA_DOWN4
ENSG00000138131	LOXL4	RNA_DOWN4
ENSG00000139679	LPAR6	RNA_DOWN4
ENSG00000117114	LPHN2	RNA_DOWN4
ENSG00000119681	LTBP2	RNA_DOWN4
ENSG00000168056	LTBP3	RNA_DOWN4
ENSG00000139329	LUM	RNA_DOWN4
ENSG00000079257	LXN	RNA_DOWN4
ENSG00000155130	MARCKS	RNA_DOWN4
ENSG00000166482	MFAP4	RNA_DOWN4
ENSG00000150051	MKX	RNA_DOWN4
ENSG00000079931	MOXD1	RNA_DOWN4
ENSG00000162576	MXRA8	RNA_DOWN4
ENSG00000176658	MYO1D	RNA_DOWN4
ENSG00000138119	MYOF	RNA_DOWN4
ENSG00000104419	NDRG1	RNA_DOWN4
ENSG00000172260	NEGR1	RNA_DOWN4
ENSG00000176046	NUPR1	RNA_DOWN4
ENSG00000116774	OLFML3	RNA_DOWN4
ENSG00000164830	OXR1	RNA_DOWN4
ENSG00000138735	PDE5A	RNA_DOWN4
ENSG00000163110	PDLIM5	RNA_DOWN4
ENSG00000118762	PKD2	RNA_DOWN4
ENSG00000102007	PLP2	RNA_DOWN4
ENSG00000164099	PRSS12	RNA_DOWN4
ENSG00000204941	PSG5	RNA_DOWN4
ENSG00000095303	PTGS1	RNA_DOWN4
ENSG00000169213	RAB3B	RNA_DOWN4
ENSG00000068028	RASSF1	RNA_DOWN4
ENSG00000142552	RCN3	RNA_DOWN4
ENSG00000121039	RDH10	RNA_DOWN4
ENSG00000122707	RECK	RNA_DOWN4
ENSG00000117152	RGS4	RNA_DOWN4
ENSG00000205413	SAMD9	RNA_DOWN4
ENSG00000139218	SCAF11	RNA_DOWN4
ENSG00000149131	SERPING1	RNA_DOWN4
ENSG00000104332	SFRP1	RNA_DOWN4
ENSG00000185960	SHOX	RNA_DOWN4
ENSG00000138771	SHROOM3	RNA_DOWN4
ENSG00000112246	SIM1	RNA_DOWN4
ENSG00000198053	SIRPA	RNA_DOWN4
ENSG00000169507	SLC38A11	RNA_DOWN4
ENSG00000145147	SLIT2	RNA_DOWN4
ENSG00000123096	SSPN	RNA_DOWN4
ENSG00000117155	SSX2IP	RNA_DOWN4
ENSG00000143028	SYPL2	RNA_DOWN4
ENSG00000204219	TCEA3	RNA_DOWN4
ENSG00000120708	TGFBI	RNA_DOWN4
ENSG00000102265	TIMP1	RNA_DOWN4
ENSG00000147027	TMEM47	RNA_DOWN4
ENSG00000133687	TMTC1	RNA_DOWN4
ENSG00000079308	TNS1	RNA_DOWN4
ENSG00000141510	TP53	RNA_DOWN4
ENSG00000175274	TP53I11	RNA_DOWN4
ENSG00000132274	TRIM22	RNA_DOWN4
ENSG00000168234	TTC39C	RNA_DOWN4
ENSG00000206538	VGLL3	RNA_DOWN4
ENSG00000164253	WDR41	RNA_DOWN4
ENSG00000173517		RNA_DOWN4
ENSG00000162688	AGL	RNA_DOWN5
ENSG00000196975	ANXA4	RNA_DOWN5
ENSG00000058668	ATP2B4	RNA_DOWN5
ENSG00000081923	ATP8B1	RNA_DOWN5
ENSG00000122786	CALD1	RNA_DOWN5
ENSG00000145349	CAMK2D	RNA_DOWN5
ENSG00000000971	CFH	RNA_DOWN5
ENSG00000164442	CITED2	RNA_DOWN5
ENSG00000082438	COBLL1	RNA_DOWN5
ENSG00000155016	CYP2U1	RNA_DOWN5
ENSG00000153071	DAB2	RNA_DOWN5
ENSG00000244274	DBNDD2	RNA_DOWN5
ENSG00000162616	DNAJB4	RNA_DOWN5
ENSG00000136048	DRAM1	RNA_DOWN5
ENSG00000177106	EPS8L2	RNA_DOWN5
ENSG00000091831	ESR1	RNA_DOWN5
ENSG00000154511	FAM69A	RNA_DOWN5
ENSG00000140092	FBLN5	RNA_DOWN5
ENSG00000138675	FGF5	RNA_DOWN5
ENSG00000155816	FMN2	RNA_DOWN5
ENSG00000179163	FUCA1	RNA_DOWN5
ENSG00000136235	GPNMB	RNA_DOWN5
ENSG00000164294	GPX8	RNA_DOWN5
ENSG00000170899	GSTA4	RNA_DOWN5
ENSG00000004776	HSPB6	RNA_DOWN5
ENSG00000100196	KDELR3	RNA_DOWN5
ENSG00000144320	KIAA1715	RNA_DOWN5
ENSG00000049130	KITLG	RNA_DOWN5
ENSG00000182195	LDOC1	RNA_DOWN5
ENSG00000107798	LIPA	RNA_DOWN5
ENSG00000113083	LOX	RNA_DOWN5
ENSG00000087253	LPCAT2	RNA_DOWN5
ENSG00000140545	MFGE8	RNA_DOWN5
ENSG00000151690	MFSD6	RNA_DOWN5
ENSG00000153029	MR1	RNA_DOWN5
ENSG00000166912	MTMR10	RNA_DOWN5
ENSG00000136783	NIPSNAP3A	RNA_DOWN5
ENSG00000122884	P4HA1	RNA_DOWN5
ENSG00000129116	PALLD	RNA_DOWN5
ENSG00000145730	PAM	RNA_DOWN5
ENSG00000112378	PERP	RNA_DOWN5
ENSG00000117592	PRDX6	RNA_DOWN5
ENSG00000139174	PRICKLE1	RNA_DOWN5
ENSG00000159200	RCAN1	RNA_DOWN5
ENSG00000164292	RHOBTB3	RNA_DOWN5
ENSG00000181852	RNF41	RNA_DOWN5
ENSG00000169432	SCN9A	RNA_DOWN5
ENSG00000080546	SESN1	RNA_DOWN5
ENSG00000168679	SLC16A4	RNA_DOWN5
ENSG00000119899	SLC17A5	RNA_DOWN5
ENSG00000106688	SLC1A1	RNA_DOWN5
ENSG00000111371	SLC38A1	RNA_DOWN5
ENSG00000095383	TBC1D2	RNA_DOWN5
ENSG00000181458	TMEM45A	RNA_DOWN5
ENSG00000067167	TRAM1	RNA_DOWN5
ENSG00000145390	USP53	RNA_DOWN5
ENSG00000103175	WFDC1	RNA_DOWN5
ENSG00000172667	ZMAT3	RNA_DOWN5
ENSG00000131584	ACAP3	P_UP1
ENSG00000189046	ALKBH2	P_UP1
ENSG00000102409	BEX4	P_UP1
ENSG00000139620	C12orf41	P_UP1
ENSG00000170270	C14orf142	P_UP1
ENSG00000176438	C14orf49	P_UP1
ENSG00000140688	C16orf58	P_UP1
ENSG00000152242	C18orf25	P_UP1
ENSG00000142686	C1orf216	P_UP1
ENSG00000125843	C20orf29	P_UP1
ENSG00000176731	C8orf59	P_UP1
ENSG00000120860	CCDC53	P_UP1
ENSG00000163660	CCNL1	P_UP1
ENSG00000163249	CCNYL1	P_UP1
ENSG00000072609	CHFR	P_UP1
ENSG00000079432	CIC	P_UP1
ENSG00000108797	CNTNAP1	P_UP1
ENSG00000130635	COL5A1	P_UP1
ENSG00000163626	COX18	P_UP1
ENSG00000160741	CRTC2	P_UP1
ENSG00000040531	CTNS	P_UP1
ENSG00000132199	ENOSF1	P_UP1
ENSG00000132635	FAM113A	P_UP1
ENSG00000039523	FAM65A	P_UP1
ENSG00000176973	FAM89B	P_UP1
ENSG00000187239	FNBP1	P_UP1
ENSG00000146830	GIGYF1	P_UP1
ENSG00000108262	GIT1	P_UP1
ENSG00000136935	GOLGA1	P_UP1
ENSG00000132522	GPS2	P_UP1
ENSG00000010704	HFE	P_UP1
ENSG00000213859	KCTD11	P_UP1
ENSG00000109787	KLF3	P_UP1
ENSG00000130702	LAMA5	P_UP1
ENSG00000091136	LAMB1	P_UP1
ENSG00000063322	MED29	P_UP1
ENSG00000240972	MIF	P_UP1
ENSG00000038945	MSR1	P_UP1
ENSG00000187193	MT1X	P_UP1
ENSG00000162601	MYSM1	P_UP1
ENSG00000132688	NES	P_UP1
ENSG00000197696	NMB	P_UP1
ENSG00000173614	NMNAT1	P_UP1
ENSG00000125741	OPA3	P_UP1
ENSG00000133027	PEMT	P_UP1
ENSG00000204138	PHACTR4	P_UP1
ENSG00000168734	PKIG	P_UP1
ENSG00000241839	PLEKHO2	P_UP1
ENSG00000143157	POGK	P_UP1
ENSG00000197562	RAB40C	P_UP1
ENSG00000169750	RAC3	P_UP1
ENSG00000123728	RAP2C	P_UP1
ENSG00000157110	RBPMS	P_UP1
ENSG00000140983	RHOT2	P_UP1
ENSG00000179833	SERTAD2	P_UP1
ENSG00000157933	SKI	P_UP1
ENSG00000225697	SLC26A6	P_UP1
ENSG00000080189	SLC35C2	P_UP1
ENSG00000143553	SNAPIN	P_UP1
ENSG00000187678	SPRY4	P_UP1
ENSG00000084112	SSH1	P_UP1
ENSG00000172830	SSH3	P_UP1
ENSG00000131748	STARD3	P_UP1
ENSG00000118046	STK11	P_UP1
ENSG00000178726	THBD	P_UP1
ENSG00000109066	TMEM104	P_UP1
ENSG00000132406	TMEM128	P_UP1
ENSG00000142188	TMEM50B	P_UP1
ENSG00000102871	TRADD	P_UP1
ENSG00000037042	TUBG2	P_UP1
ENSG00000105397	TYK2	P_UP1
ENSG00000158773	USF1	P_UP1
ENSG00000123154	WDR83	P_UP1
ENSG00000171163	ZNF692	P_UP1
ENSG00000223526		P_UP1
ENSG00000158669	AGPAT6	P_UP2
ENSG00000170017	ALCAM	P_UP2
ENSG00000154188	ANGPT1	P_UP2
ENSG00000180801	ARSJ	P_UP2
ENSG00000164291	ARSK	P_UP2
ENSG00000104763	ASAH1	P_UP2
ENSG00000164122	ASB5	P_UP2
ENSG00000068650	ATP11A	P_UP2
ENSG00000058063	ATP11B	P_UP2
ENSG00000081923	ATP8B1	P_UP2
ENSG00000121578	B4GALT4	P_UP2
ENSG00000106392	C1GALT1	P_UP2
ENSG00000115355	CCDC88A	P_UP2
ENSG00000172831	CES2	P_UP2
ENSG00000169826	CSGALNACT2	P_UP2
ENSG00000101441	CST4	P_UP2
ENSG00000154639	CXADR	P_UP2
ENSG00000162144	CYBASC3	P_UP2
ENSG00000077232	DNAJC10	P_UP2
ENSG00000171451	DSEL	P_UP2
ENSG00000182585	EPGN	P_UP2
ENSG00000068912	ERLEC1	P_UP2
ENSG00000136068	FLNB	P_UP2
ENSG00000125848	FLRT3	P_UP2
ENSG00000164930	FZD6	P_UP2
ENSG00000153982	GDPD1	P_UP2
ENSG00000111670	GNPTAB	P_UP2
ENSG00000142166	IFNAR1	P_UP2
ENSG00000134352	IL6ST	P_UP2
ENSG00000129636	ITFG1	P_UP2
ENSG00000115919	KYNU	P_UP2
ENSG00000205213	LGR4	P_UP2
ENSG00000164187	LMBRD2	P_UP2
ENSG00000173114	LRRN3	P_UP2
ENSG00000112893	MAN2A1	P_UP2
ENSG00000105976	MET	P_UP2
ENSG00000168282	MGAT2	P_UP2
ENSG00000160588	MPZL3	P_UP2
ENSG00000070614	NDST1	P_UP2
ENSG00000091039	OSBPL8	P_UP2
ENSG00000160613	PCSK7	P_UP2
ENSG00000145431	PDGFC	P_UP2
ENSG00000006576	PHTF2	P_UP2
ENSG00000197563	PIGN	P_UP2
ENSG00000174136	RGMB	P_UP2
ENSG00000170881	RNF139	P_UP2
ENSG00000025796	SEC63	P_UP2
ENSG00000149577	SIDT2	P_UP2
ENSG00000168575	SLC20A2	P_UP2
ENSG00000145740	SLC30A5	P_UP2
ENSG00000162695	SLC30A7	P_UP2
ENSG00000117620	SLC35A3	P_UP2
ENSG00000157800	SLC37A3	P_UP2
ENSG00000111371	SLC38A1	P_UP2
ENSG00000033867	SLC4A7	P_UP2
ENSG00000102359	SRPX2	P_UP2
ENSG00000163527	STT3B	P_UP2
ENSG00000075568	TMEM131	P_UP2
ENSG00000164168	TMEM184C	P_UP2
ENSG00000135048	TMEM2	P_UP2
ENSG00000109133	TMEM33	P_UP2
ENSG00000153214	TMEM87B	P_UP2
ENSG00000139324	TMTC3	P_UP2
ENSG00000163728	TTC14	P_UP2
ENSG00000052841	TTC17	P_UP2
ENSG00000148154	UGCG	P_UP2
ENSG00000150630	VEGFC	P_UP2
ENSG00000128564	VGF	P_UP2
ENSG00000178381	ZFAND2A	P_UP2
ENSG00000143401	ANP32E	P_DOWN1
ENSG00000106635	BCL7B	P_DOWN1
ENSG00000163170	BOLA3	P_DOWN1
ENSG00000164967	C9orf23	P_DOWN1
ENSG00000169217	CD2BP2	P_DOWN1
ENSG00000203760	CENPW	P_DOWN1
ENSG00000173207	CKS1B	P_DOWN1
ENSG00000127184	COX7C	P_DOWN1
ENSG00000176340	COX8A	P_DOWN1
ENSG00000157326	DHRS4	P_DOWN1
ENSG00000149636	DSN1	P_DOWN1
ENSG00000114942	EEF1B2	P_DOWN1
ENSG00000100129	EIF3L	P_DOWN1
ENSG00000132507	EIF5A	P_DOWN1
ENSG00000120533	ENY2	P_DOWN1
ENSG00000035141	FAM136A	P_DOWN1
ENSG00000166797	FAM96A	P_DOWN1
ENSG00000167807	FDX1L	P_DOWN1
ENSG00000088832	FKBP1A	P_DOWN1
ENSG00000133393	FOPNL	P_DOWN1
ENSG00000109536	FRG1	P_DOWN1
ENSG00000156958	GALK2	P_DOWN1
ENSG00000152147	GEMIN6	P_DOWN1
ENSG00000174021	GNG5	P_DOWN1
ENSG00000233276	GPX1	P_DOWN1
ENSG00000184897	H1FX	P_DOWN1
ENSG00000134248	HBXIP	P_DOWN1
ENSG00000205581	HMGN1	P_DOWN1
ENSG00000135486	HNRNPA1	P_DOWN1
ENSG00000139675	HNRNPA1L2	P_DOWN1
ENSG00000081870	HSPB11	P_DOWN1
ENSG00000155090	KLF10	P_DOWN1
ENSG00000130520	LSM4	P_DOWN1
ENSG00000162441	LZIC	P_DOWN1
ENSG00000111196	MAGOHB	P_DOWN1
ENSG00000198042	MAK16	P_DOWN1
ENSG00000037897	METTL1	P_DOWN1
ENSG00000135698	MPHOSPH6	P_DOWN1
ENSG00000180992	MRPL14	P_DOWN1
ENSG00000182154	MRPL41	P_DOWN1
ENSG00000136371	MTHFS	P_DOWN1
ENSG00000152082	MZT2B	P_DOWN1
ENSG00000128534	NAA38	P_DOWN1
ENSG00000145494	NDUFS6	P_DOWN1
ENSG00000213281	NRAS	P_DOWN1
ENSG00000105185	PDCD5	P_DOWN1
ENSG00000100410	PHF5A	P_DOWN1
ENSG00000115946	PNO1	P_DOWN1
ENSG00000186184	POLR1D	P_DOWN1
ENSG00000105258	POLR2I	P_DOWN1
ENSG00000147669	POLR2K	P_DOWN1
ENSG00000132963	POMP	P_DOWN1
ENSG00000131795	RBM8A	P_DOWN1
ENSG00000204308	RNF5	P_DOWN1
ENSG00000142676	RPL11	P_DOWN1
ENSG00000142541	RPL13A	P_DOWN1
ENSG00000105640	RPL18A	P_DOWN1
ENSG00000122026	RPL21	P_DOWN1
ENSG00000116251	RPL22	P_DOWN1
ENSG00000125691	RPL23	P_DOWN1
ENSG00000161970	RPL26	P_DOWN1
ENSG00000131469	RPL27	P_DOWN1
ENSG00000100316	RPL3	P_DOWN1
ENSG00000071082	RPL31	P_DOWN1
ENSG00000144713	RPL32	P_DOWN1
ENSG00000109475	RPL34	P_DOWN1
ENSG00000136942	RPL35	P_DOWN1
ENSG00000241343	RPL36A	P_DOWN1
ENSG00000145592	RPL37	P_DOWN1
ENSG00000174444	RPL4	P_DOWN1
ENSG00000229117	RPL41	P_DOWN1
ENSG00000122406	RPL5	P_DOWN1
ENSG00000089009	RPL6	P_DOWN1
ENSG00000147604	RPL7	P_DOWN1
ENSG00000163682	RPL9	P_DOWN1
ENSG00000177600	RPLP2	P_DOWN1
ENSG00000142534	RPS11	P_DOWN1
ENSG00000112306	RPS12	P_DOWN1
ENSG00000164587	RPS14	P_DOWN1
ENSG00000134419	RPS15A	P_DOWN1
ENSG00000105193	RPS16	P_DOWN1
ENSG00000231500	RPS18	P_DOWN1
ENSG00000105372	RPS19	P_DOWN1
ENSG00000186468	RPS23	P_DOWN1
ENSG00000177954	RPS27	P_DOWN1
ENSG00000213741	RPS29	P_DOWN1
ENSG00000149273	RPS3	P_DOWN1
ENSG00000145425	RPS3A	P_DOWN1
ENSG00000198034	RPS4X	P_DOWN1
ENSG00000137154	RPS6	P_DOWN1
ENSG00000171863	RPS7	P_DOWN1
ENSG00000142937	RPS8	P_DOWN1
ENSG00000184990	SIVA1	P_DOWN1
ENSG00000169100	SLC25A6	P_DOWN1
ENSG00000152253	SPC25	P_DOWN1
ENSG00000169689	STRA13	P_DOWN1
ENSG00000188612	SUMO2	P_DOWN1
ENSG00000171530	TBCA	P_DOWN1
ENSG00000126768	TIMM17B	P_DOWN1
ENSG00000126953	TIMM8A	P_DOWN1
ENSG00000075131	TIPIN	P_DOWN1
ENSG00000133112	TPT1	P_DOWN1
ENSG00000173113	TRMT112	P_DOWN1
ENSG00000136810	TXN	P_DOWN1
ENSG00000100749	VRK1	P_DOWN1
ENSG00000065978	YBX1	P_DOWN1
ENSG00000104231	ZFAND1	P_DOWN1
ENSG00000255432		P_DOWN1
ENSG00000033050	ABCF2	P_DOWN2
ENSG00000129055	ANAPC13	P_DOWN2
ENSG00000149089	APIP	P_DOWN2
ENSG00000145782	ATG12	P_DOWN2
ENSG00000105341	ATP5SL	P_DOWN2
ENSG00000105583	C19orf56	P_DOWN2
ENSG00000143751	C1orf55	P_DOWN2
ENSG00000164885	CDK5	P_DOWN2
ENSG00000143942	CHAC2	P_DOWN2
ENSG00000166200	COPS2	P_DOWN2
ENSG00000111481	COPZ1	P_DOWN2
ENSG00000133983	COX16	P_DOWN2
ENSG00000153015	CWC27	P_DOWN2
ENSG00000130311	DDA1	P_DOWN2
ENSG00000178105	DDX10	P_DOWN2
ENSG00000139726	DENR	P_DOWN2
ENSG00000175550	DRAP1	P_DOWN2
ENSG00000198692	EIF1AY	P_DOWN2
ENSG00000063046	EIF4B	P_DOWN2
ENSG00000119616	FCF1	P_DOWN2
ENSG00000114416	FXR1	P_DOWN2
ENSG00000109534	GAR1	P_DOWN2
ENSG00000242616	GNG10	P_DOWN2
ENSG00000164284	GRPEL2	P_DOWN2
ENSG00000164032	H2AFZ	P_DOWN2
ENSG00000130021	HDHD1	P_DOWN2
ENSG00000176407	KCMF1	P_DOWN2
ENSG00000188223	LIN37	P_DOWN2
ENSG00000148943	LIN7C	P_DOWN2
ENSG00000139233	LLPH	P_DOWN2
ENSG00000175324	LSM1	P_DOWN2
ENSG00000120992	LYPLA1	P_DOWN2
ENSG00000198015	MRPL42	P_DOWN2
ENSG00000136522	MRPL47	P_DOWN2
ENSG00000147586	MRPS28	P_DOWN2
ENSG00000214114	MYCBP	P_DOWN2
ENSG00000109065	NAT9	P_DOWN2

Table S2

ENSG00000225921	NOL7	P_DOWN2
ENSG00000164346	NSA2	P_DOWN2
ENSG00000139579	OBFC2B	P_DOWN2
ENSG00000172239	PAIP1	P_DOWN2
ENSG00000101132	PFDN4	P_DOWN2
ENSG00000164902	PHAX	P_DOWN2
ENSG00000106588	PSMA2	P_DOWN2
ENSG00000168118	RAB4A	P_DOWN2
ENSG00000144840	RABL3	P_DOWN2
ENSG00000089050	RBBP9	P_DOWN2
ENSG00000108298	RPL19	P_DOWN2
ENSG00000114391	RPL24	P_DOWN2
ENSG00000166441	RPL27A	P_DOWN2
ENSG00000162244	RPL29	P_DOWN2
ENSG00000156482	RPL30	P_DOWN2
ENSG00000182899	RPL35A	P_DOWN2
ENSG00000172809	RPL38	P_DOWN2
ENSG00000198918	RPL39	P_DOWN2
ENSG00000146223	RPL7L1	P_DOWN2
ENSG00000110700	RPS13	P_DOWN2
ENSG00000138326	RPS24	P_DOWN2
ENSG00000143947	RPS27A	P_DOWN2
ENSG00000129824	RPS4Y1	P_DOWN2
ENSG00000137876	RSL24D1	P_DOWN2
ENSG00000163156	SCNM1	P_DOWN2
ENSG00000052723	SIKE1	P_DOWN2
ENSG00000174446	SNAPC5	P_DOWN2
ENSG00000131876	SNRPA1	P_DOWN2
ENSG00000182004	SNRPE	P_DOWN2
ENSG00000139343	SNRPF	P_DOWN2
ENSG00000123178	SPRYD7	P_DOWN2
ENSG00000172070	SRXN1	P_DOWN2
ENSG00000116030	SUMO1	P_DOWN2
ENSG00000154582	TCEB1	P_DOWN2
ENSG00000051596	THOC3	P_DOWN2
ENSG00000163634	THOC7	P_DOWN2
ENSG00000116918	TSNAX	P_DOWN2
ENSG00000115514	TXNDC9	P_DOWN2
ENSG00000164405	UQCRQ	P_DOWN2
ENSG00000103194	USP10	P_DOWN2
ENSG00000132467	UTP3	P_DOWN2
ENSG00000120963	ZNF706	P_DOWN2
ENSG00000108278	ZNHIT3	P_DOWN2
ENSG00000259399		P_DOWN2
